# Design, Synthesis, Pharmacodynamic and *In Silico* Pharmacokinetic Evaluation of Some Novel Biginelli-Derived Pyrimidines and Fused Pyrimidines as Calcium Channel Blockers

**DOI:** 10.3390/molecules27072240

**Published:** 2022-03-30

**Authors:** Ahmed M. Farghaly, Ola H. Rizk, Inas Darwish, Manal Hamza, Mezna Saleh Altowyan, Assem Barakat, Mohamed Teleb

**Affiliations:** 1 Department of Pharmaceutical Chemistry, Faculty of Pharmacy, Alexandria University, Alexandria 21521, Egypt; olarizk@yahoo.com; 2 Department of Pharmaceutical Chemistry, Faculty of Pharmacy, Pharos University, Alexandria 21521, Egypt; 3 Department of Clinical Pharmacology, Faculty of Medicine, Alexandria University, Alexandria 21521, Egypt; inas.darwish@alexmed.edu.eg (I.D.); manalhamza@gmail.com (M.H.); 4 Department of Pharmacology and Therapeutics, Faculty of Pharmacy, Pharos University, Alexandria 21521, Egypt; 5 Department of Chemistry, College of Science, Princess Nourah bint Abdulrahman University, P.O. Box 84428, Riyadh 11671, Saudi Arabia; msaltowyan@pnu.edu.sa; 6 Department of Chemistry, College of Science, King Saud University, P.O. Box 2455, Riyadh 11451, Saudi Arabia; 7 Department of Chemistry, Faculty of Science, Alexandria University, P.O. Box 426, Ibrahimia, Alexandria 21321, Egypt

**Keywords:** pyrimidines, Biginelli, calcium channel blockers, hypotensive activity

## Abstract

Some new pyrimidine derivatives comprising arylsulfonylhydrazino, ethoxycarbonylhydrazino, thiocarbamoylhydrazino and substituted hydrazone and thiosemicarbazide functionalities were prepared from Biginelli-derived pyrimidine precursors. Heterocyclic ring systems such as pyrazole, pyrazolidinedione, thiazoline and thiazolidinone ring systems were also incorporated into the designed pyrimidine core. Furthermore, fused triazolopyrimidine and pyrimidotriazine ring systems were prepared. The synthesized compounds were evaluated for their calcium channel blocking activity as potential hypotensive agents. Compounds **2**, **3a**, **3b**, **4**, **11** and **13** showed the highest ex vivo calcium channel blocking activities compared with the reference drug nifedipine. Compounds **2** and **11** were selected for further biological evaluation. They revealed good hypotensive activities following intravenous administration in dogs. Furthermore, **2** and **11** displayed drug-like *in silico* ADME parameters. A ligand-based pharmacophore model was developed to provide adequate information about the binding mode of the newly synthesized active compounds **2**, **3a**, **3b**, **4**, **11** and **13**. This may also serve as a reliable basis for designing new active pyrimidine-based calcium channel blockers.

## 1. Introduction

The American Heart Association reported an average of one death every 40 s due to cardiovascular diseases (CVDs) [1]. The WHO Global Atlas on Cardiovascular Disease Prevention and Control confirmed that CVDs are the leading cause of mortality worldwide [2]. In response to the burden posed by CVDs, the European Society of Cardiology (ESC) and the European Society of Hypertension (ESH) published their guidelines and recommended several cardiovascular agents in the clinic. However, in many cases, their clinical use is limited by their side effects [3]. Therefore, there is a continuous need for developing novel efficient cardiovascular agents.

**Figure 1:**
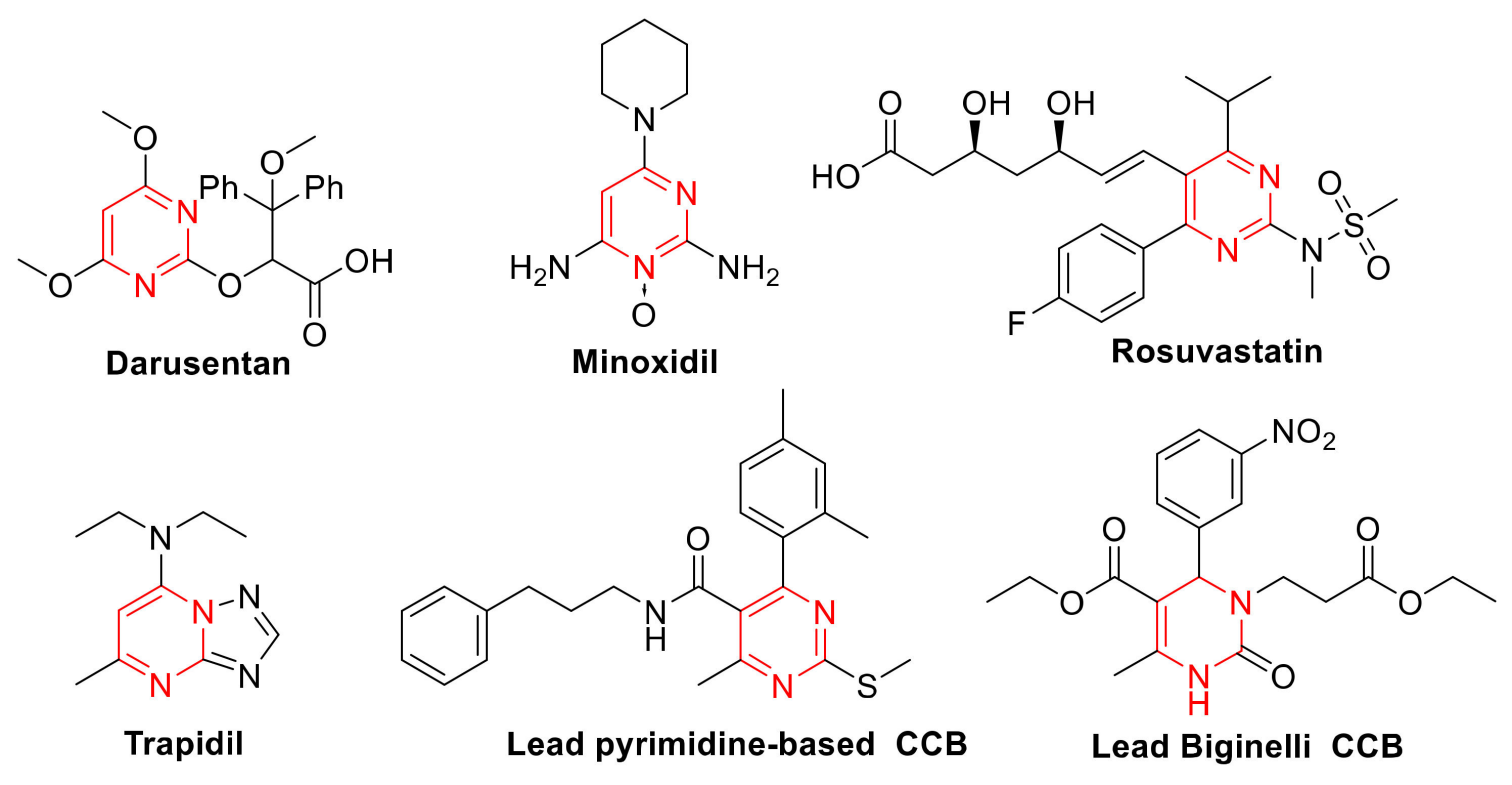
Pyrimidine-derived cardiovascular agents.

Based on their chemical structures, many cardiovascular agents are pyrimidine derivatives (Figure 1), such as darusentan, a selective endothelin receptor antagonist [4]; minoxidil, a direct vasodilator [5]; rosuvastatin, a competitive inhibitor of HMG-CoA reductase [6]; and trapidil, a fused pyrimidine vasodilator [7]. Extensive exploration of the pyrimidine ring system led to the synthesis of novel orally active angiotensin II antagonists [8] and efficient calcium channel blockers (CCBs) which gained considerable interest [9]. Such a large representation of this heterocyclic nucleus in cardiovascular agents suggests that this heterocyclic moiety, if properly decorated with substituents, could lead to novel potential cardiovascular agents.

**Figure 2:**
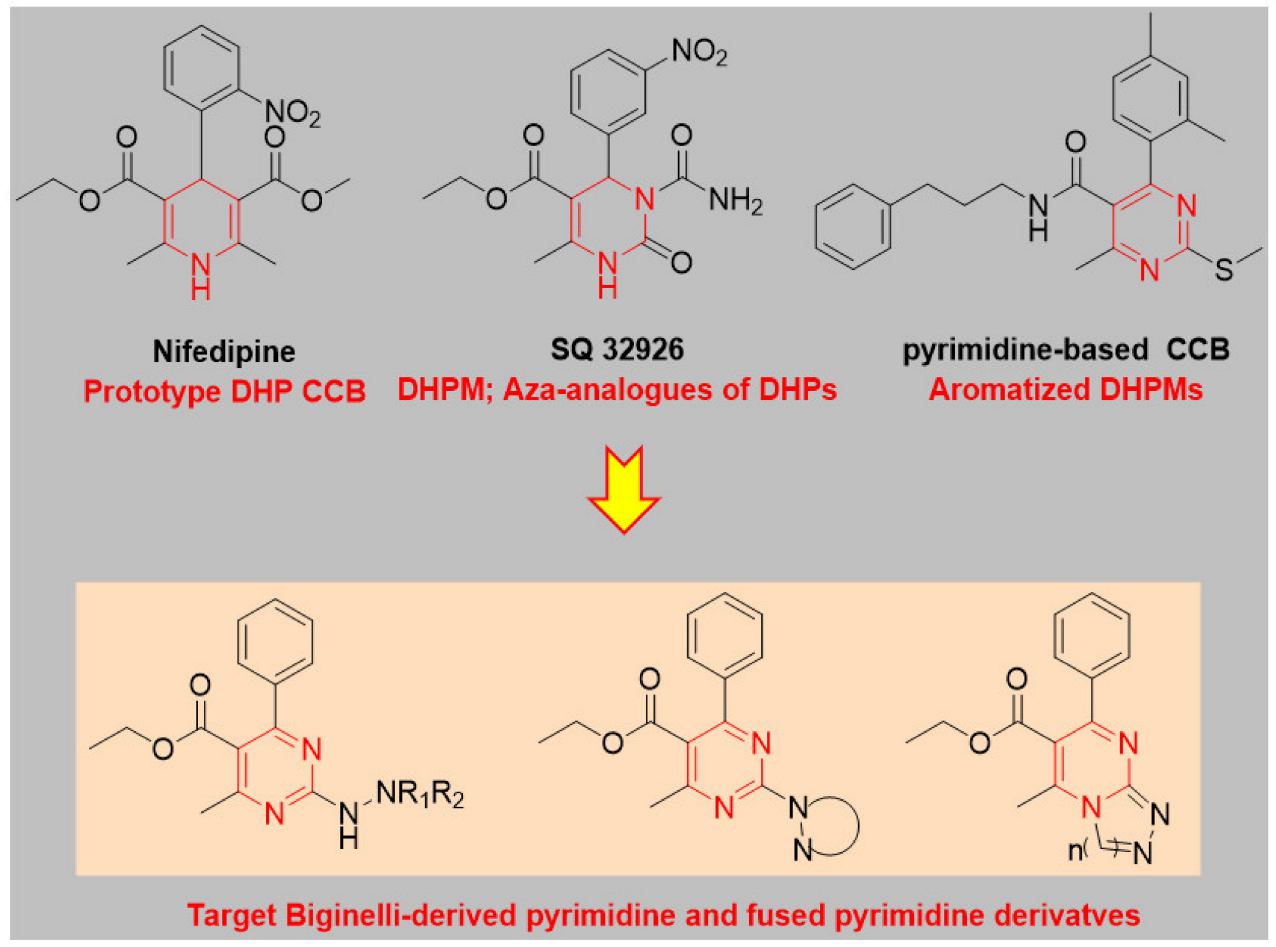
The design strategy of the target Biginelli-derived pyrimidines and fused pyrimidines.

Here we report the synthesis of novel pyrimidine derivatives (Figure 2) designed by taking advantage of the high diversity initially generated on the pyrimidine core through Biginelli multicomponent reaction [10]. Inspired by the Biginelli-derived CCBs that are considered aza-analogs of dihydropyridine (DHP) CCBs [11,12,13,14,15] (Figure 2), the newly synthesized derivatives were rationalized as potential CCBs. This hypothesis was also supported by the representation of the pyrimidine ring in efficient CCBs [9]. Accordingly, all the target compounds were evaluated for their in vitro calcium channel blocking activity relative to the prototype CCB nifedipine. The most active derivatives were then evaluated for possible hypotensive activities in dogs, then subjected to molecular modeling studies. The substitution pattern was rationalized to keep the basic pharmacophoric core while modifying the C^2^ position. In this regard, various alkyl and aryl moieties were introduced at the core’s C^2^ via different functionalized linkers (hydrazones, thiosemicarbazides, etc.) following the SAR of previously reported CCBs [12,13,14,15]. Heterocyclic ring systems such as pyrazole, pyrazolidinedione, thiazoline and thiazolidinone were also incorporated. Furthermore, it was also aimed to synthesize fused pyrimidine ring systems such as triazolopyrimidine and pyrimidotriazine rings to extend the deduced structure–activity relationship study. It is worth mentioning that these nitrogenous heterocycles are obviously represented in various lead CCBs [12,13,14,15,16,17,18]. 

The synthesized pyrimidine and fused pyrimidine derivatives were evaluated for their potential calcium channel blocking activity as hypotensive agents. The compounds showing promising ex vivo calcium channel blocking activities were then tested for their hypotensive activity following intravenous administration in dogs. Nifedipine was selected as a reference as it is the prototype DHP CCB and the lead for Biginelli-derived dihydropyrimidine (DHPM) CCBs and pyrimidine-based CCBs. Additionally, a ligand-based pharmacophore model was developed to provide adequate information about the binding mode of the newly synthesized active compounds. This may also serve as a reliable basis for designing new active pyrimidine-based CCBs. 

## 2. Results and Discussion

### 2.1. Chemistry

**Scheme 1:**
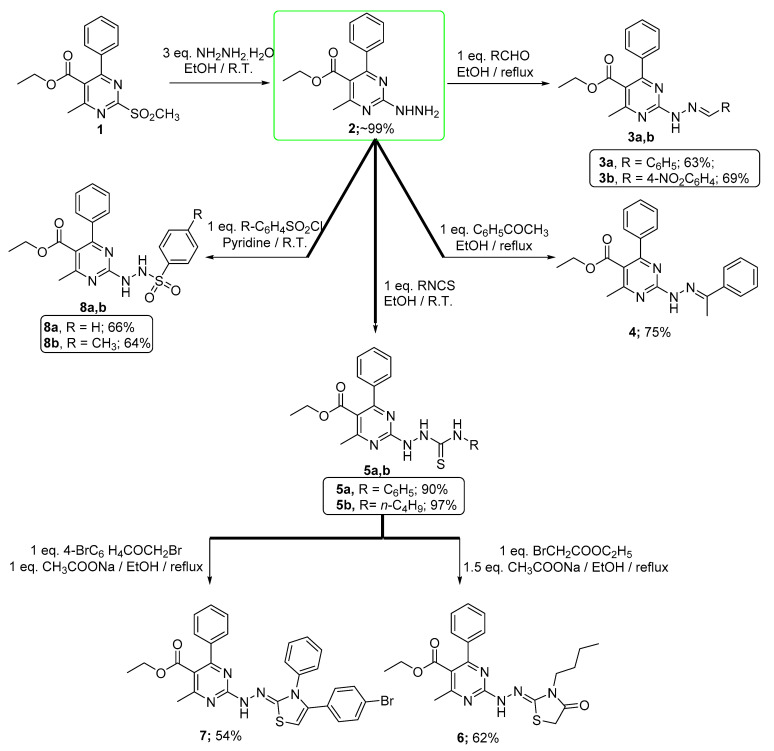
Synthesis of the desired compounds **2**–**8**.

**Scheme 2:**
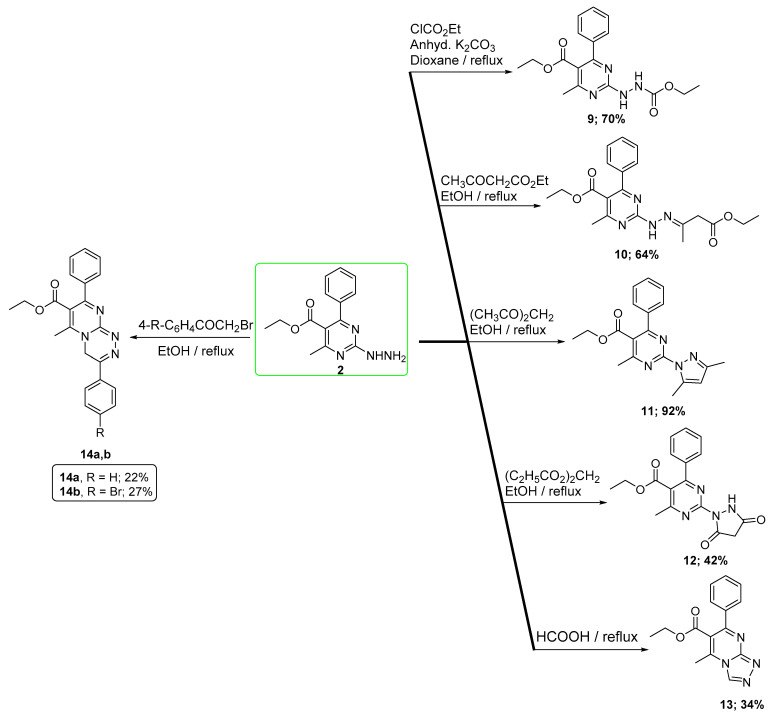
Synthesis of the desired compounds **9**–**14**.

The synthetic strategies adopted for the preparation of the intermediate and final compounds are depicted in Scheme 1 and Scheme 2. As shown in Scheme 1, the starting compound ethyl 6-methyl-2-methylsulfonyl-4-phenylpyrimidine-5-carboxylate **1** [19] was conveniently converted to ethyl 2-hydrazino-6-methyl-4-phenylpyrimidine-5-carboxylate **2** by reaction with 99% hydrazine hydrate in ethanol. ^1^H-NMR showed the absence of the methyl singlet and the appearance of the D_2_O exchangeable signals at 4.33 and 8.62 ppm assigned to NH_2_ and NH, respectively. Condensing equimolar amounts of hydrazine derivative **2** with aromatic aldehydes and acetophenone as representative ketone in refluxing EtOH following a conventional method [20] afforded the corresponding hydrazones **3a**,**b** and **4,** respectively. The IR spectra of these compounds lacked stretching absorption bands due to NH_2_ and showed stretching absorption bands due to NH and C=N, while ^1^H-NMR lacked the upfield D_2_O-exchangeable singlet assigned for hydrazine NH_2_ protons and showed a downfield D_2_O-exchangeable singlet assigned for hydrazone NH proton. The structure of compound **4** was further verified by ^13^C-NMR spectral data. The new thiosemicarbazides **5a**,**b** were prepared by reaction of the key intermediate **2** with representative aryl- and alkyl-substituted isothiocyanates at room temperature. Reaction of **5b** with ethyl bromoacetate in boiling EtOH containing anhydrous sodium acetate [21] afforded the corresponding thiazolidinone derivative **6b.** ^1^H-NMR showed a highly deshielded D_2_O-exchangeable singlet assigned for NH proton. In addition, a multiplet integrated for two protons was assigned for thiazolidinone C^5^ protons, while the ^13^C-NMR spectrum provided further confirmation of the structure. Moreover, condensing the thiosemicarbazides **5a** with bromophenacyl bromide in presence of sodium acetate in absolute EtOH [21] afforded the thiazoline derivatives **7** in acceptable yields. Its ^1^H-NMR spectrum showed a deshielded singlet, integrated for one proton assigned for thiazoline C^5^ proton. Compounds **8a**,**b** were synthesized by stirring equimolecular amounts of the hydrazine **2** with the aryl sulfonyl chlorides in dry pyridine following a previously reported procedure [22]. Products were identified by IR, ^1^H-NMR and ^13^C-NMR in addition to MS spectrum of **8b** which showed a molecular ion peak at *m*/*z* 426 (20%) that matched its molecular weight. 

Referring to Scheme 2, the key intermediate **2** was heated with ethyl chloroformate in dry dioxane in accordance with conventional procedure [23] affording ethyl 2-[2-(ethoxycarbonyl)hydrazino]-6-methyl-4-phenylpyrimidine-5-carboxylate **9**. The ^1^H-NMR spectrum showed an extra triplet and quartet characteristic of the ethyl moiety, while its MS spectrum revealed a molecular ion peak at *m*/*z* 344 (14%) which matched its molecular weight. Condensation of hydrazino derivative **2** with ethyl acetoacetate yielded the corresponding hydrazone **10**. The chemical structure of **10** was confirmed by IR, ^1^H-NMR, ^13^C-NMR and MS spectral data. The pyrazolyl derivatives **11** and **12** were successfully produced by heating **2** with acetylacetone and diethylmalonate, respectively, in ethanol/glacial acetic acid. ^1^H-NMR spectra of compounds **11** and **12** were characterized by pyrazolyl C^4^ protons. ^13^C-NMR spectra for these compounds revealed a signal at 110.80 ppm due to pyrazolyl C^4^. Moreover, the MS spectrum of **11** showed a molecular ion peak at *m*/*z* 336 (100%) which is in accordance with its molecular formula. 

**Figure 3:**
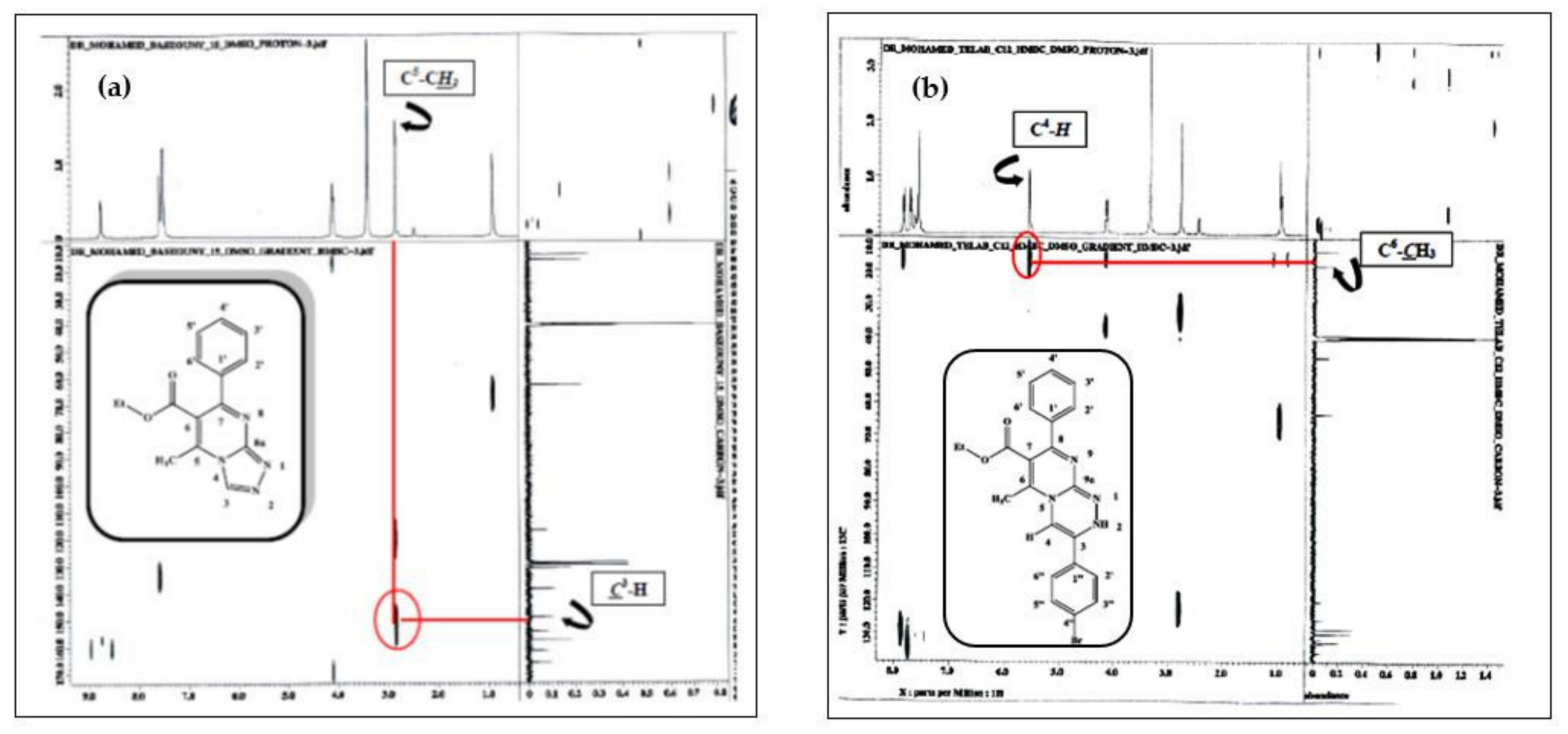
(**a**) HMBC spectrum of compound **13**; (**b**) HMBC spectrum of compound **14b**.

Heating compound **2** with formic acid [24] gave the corresponding ethyl 5-methyl-7-phenyl-1,2,4-triazolo[4,3-*a*]pyrimidine-6-carbxylate **13**. Its ^1^H-NMR spectrum lacked the two D_2_O-exchangeable singlets assigned for NHNH_2_ protons and showed a new downfield singlet assigned for triazole C_3_-H proton, confirming cyclization. Its ^13^C-NMR spectrum revealed two signals at 148.48 and 160.86 ppm corresponding to triazolopyrimidine C^3^ and C^8a^, respectively. Additionally, its MS spectrum showed a molecular ion peak at *m*/*z* 282 (54%) which is in accordance with its molecular formula. Cyclization regioselectivity of **13** was unequivocally established by HMBC showing a correlation between C^5^-CH_3_ at 2.9 ppm and the C^3^ at 148.48 ppm (Figure 3a), confirming cyclization at N^1^ rather than N^3^ of the pyrimidine core. On the other hand, compound **2** was cyclized with the appropriate phenacyl bromides in boiling absolute ethanol [25] to give the pyrimido[2,1-*c*]-1,2,4-triazine derivatives **14a**,**b**. ^1^H-NMR spectra of these compounds showed the presence of singlets at 5.54–5.56 ppm assigned to the triazino C^4^ protons. The ^13^C-NMR spectrum of compound **14b** revealed signals due to pyrimidotriazine C^3^ and C^4^ at their expected chemical shifts. Moreover, the MS spectrum of **14a** showed a molecular ion peak at *m*/*z* 372 (65%) which matched its molecular weight. Similarly, cyclization regioselectivity of **14b** was unequivocally established by HMBC showing a correlation between C^6^-CH_3_ carbon at 17.47 ppm and the C^4^-H at 5.54 ppm (Figure 3b), confirming cyclization at N^1^ rather than N^3^ of the pyrimidine core.

### 2.2. Biological Evaluation 

All the newly synthesized derivatives were screened for calcium channel blocking activity by determining their ability to antagonize KCl-induced contractions of isolated rabbit jejunum and rat colon at a concentration of 10^−5^ M [26] (Table 1). Results of the preliminary screening revealed that six compounds (**2**, **3a**, **3b**, **4**, **11** and **13**) showed inhibition of KCl-induced contractions, whereas other compounds failed to initiate any detectable activity. Candidate compounds were then evaluated at increasing doses (2 × 10^−5^, 4 × 10^−5^ and 6 × 10^−5^ M) (Table 2). Active compounds were less potent than nifedipine. However, they showed dose-dependent inhibition of KCl-induced contractions. The highest calcium channel blockade was exhibited by the hydrazine derivative **2** and the *p*-nitrophenylhydrazone **3b**. They were equipotent, showing 100% inhibition of KCl-induced contractions at a concentration of 6 × 10^−5^ M. The hydrazones **3a** and **4** lacking the nitro group showed lower activity at the same concentration. Moderate activity was exhibited when the hydrazine functionality was encaged in a planar heterocyclic pyrazole ring to furnish compound **11**. The lowest detected activity was elicited when a triazole ring was fused to the pyrimidine ring in compound **13**. For further quantitative assessment, IC_50_ and pIC_50_ were statistically calculated (Table 3). Results showed that the lead compound **2** was the most potent. The hydrazones **3a**, **3b** and **4** as well as the pyrazole derivative **11** showed moderate activities. The fused triazolopyrimidine derivative **13** showed the least calcium channel blocking activity.

In addition, compounds **2** and **11** were evaluated for hypotensive activity (mg/kg, i.v.) in normotensive anesthetized dogs at different doses [27] (Table 4). Results are represented by the change in mean arterial blood pressure (MAP) (mmHg). The data indicated a poor correlation between in vitro calcium channel blocking activity and hypotensive activity in normotensive anesthetized dogs following i.v. administration of compounds **2** and **11** at doses up to 12 mg/kg. Additional studies were performed at higher doses, where both compounds exhibited approximately the same potency at 24 mg/kg i.v. dose. 

### 2.3. Molecular Modeling

#### 2.3.1. Pharmacophore Modeling

**Figure 4:**
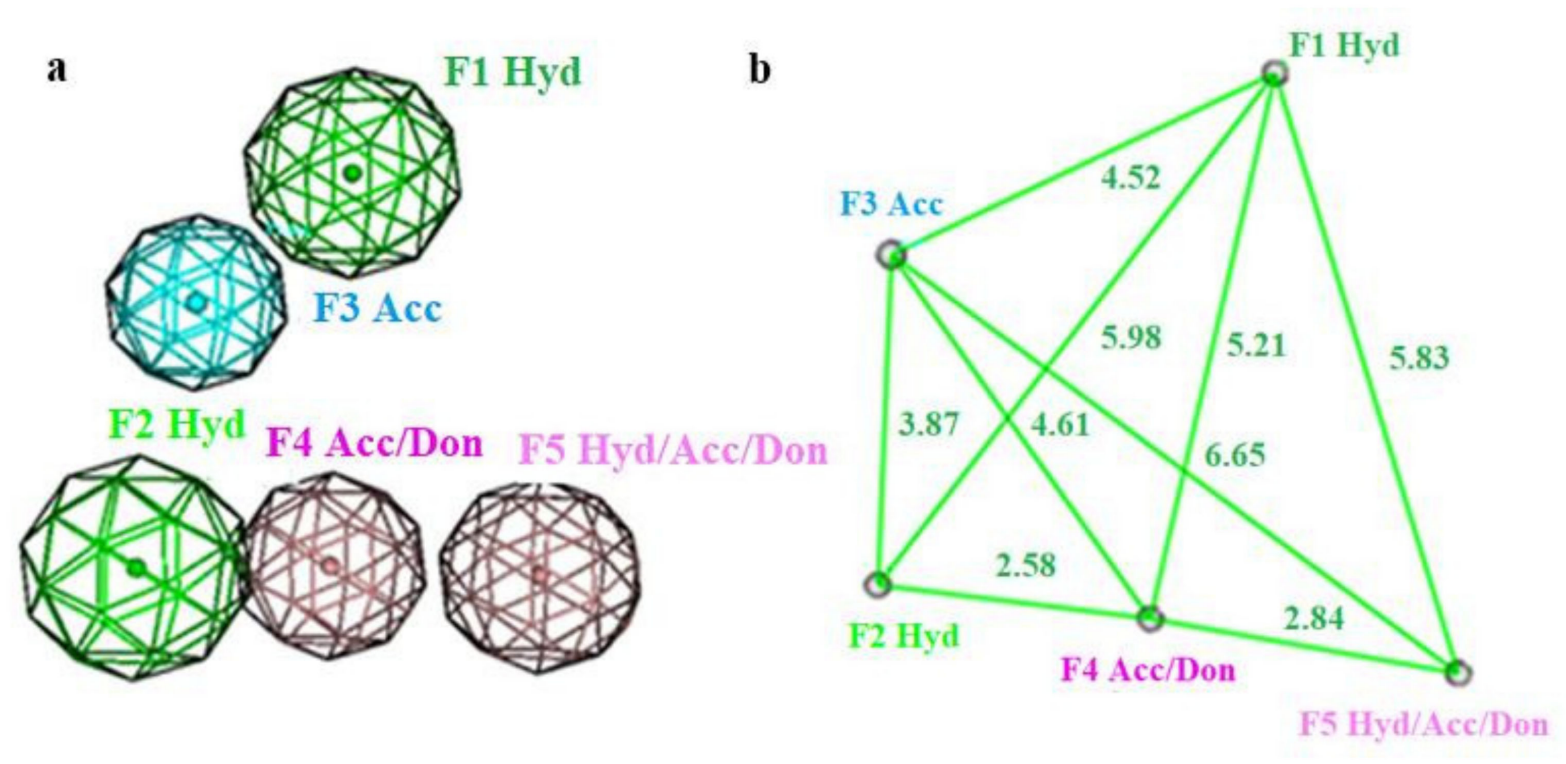
(**a**) The best query displaying pharmacophoric features shared by representative DHP, DHPM and pyrimidine-based CCBs as colored spheres (green for hydrophobic feature, cyan for H-bond acceptor and pink for H-bond acceptor/donor as well as hydrophobic centers with H-bond acceptor or donor functions. (**b**) Linear distances between various pharmacophore features are measured in angstroms and displayed as green lines.

In the present investigation, a ligand-based pharmacophore model was developed for representative DHP CCBs, including the prototype nifedipine and its lead aza-analogs; DHPM CCBs; and pyrimidine-based CCBs [9,28,29,30] as a training set (Supplementary File Figure S29) in order to map common structural features of highly active CCBs (Figure 4). 

In absence of the 3D structure of LCC, this hypothesis was employed as a valuable tool to provide adequate information about the binding mode of the newly synthesized active compounds. This may also provide a reliable basis for the design of new potentially active molecules of the pyrimidine type. All structures were built using MOE Builder in the Molecular Operating Environment program (MOE) [31]. The selected 3D-pharmacophore model (pharmacophore query) showed 100% accuracy and 7.8 overlap and was composed of five main features (Figure 4a):

1. Hydrophobic center (green sphere) involving C^4^ phenyl ring (F1: Hyd).
2. Hydrophobic center (green sphere) involving C^6^ methyl group (F2: Hyd).
3. Hydrogen bond acceptor function (cyan sphere) involving C^5^ carbonyl group (F3: Acc).
4. Hydrogen bond acceptor/donor function (pink sphere) involving ring N (F4: Acc/Don).
5. Hydrophobic center with H-bond acceptor or donor function (pink sphere) involving C^2^ substitutions (F5: Hyd/Acc/Don).

The 3D spatial relationship between these key features, identified by pharmacophore analysis, was reported as linear distances in angstroms (Figure 4b).

The selected pharmacophore model was validated for its predictive efficacy as a calcium channel model utilizing representative derivatives of DHP, DHPM [29] and pyrimidine [9] CCBs as a validation set (Supplementary File Figures S30 and S31). Biologically active compounds were subjected to conformational search and energy minimization and were superimposed onto the pharmacophore hypothesis. The most suitable alignment for each compound (lowest RMSD) was selected (Table 5).

**Figure 5:**
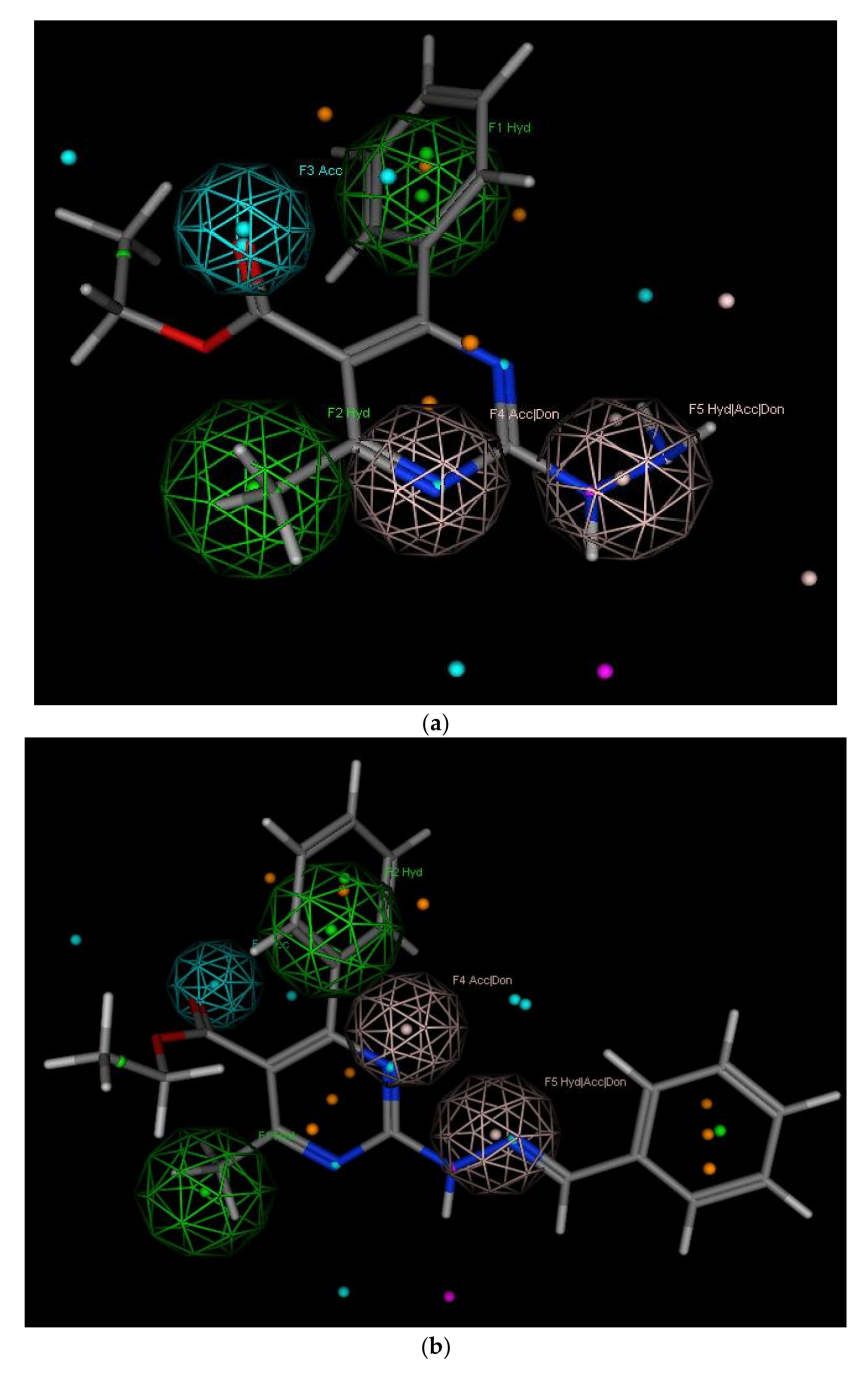

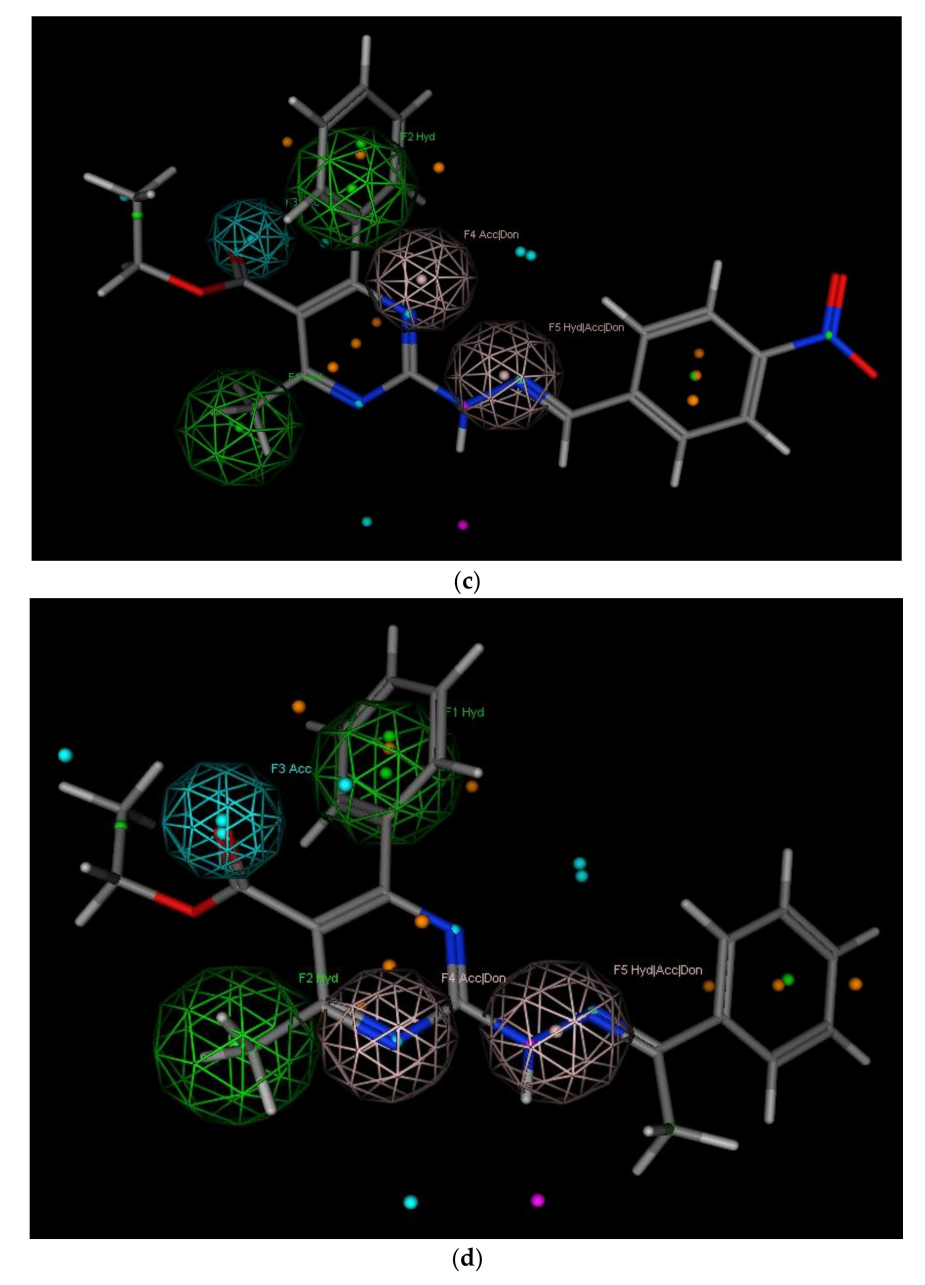

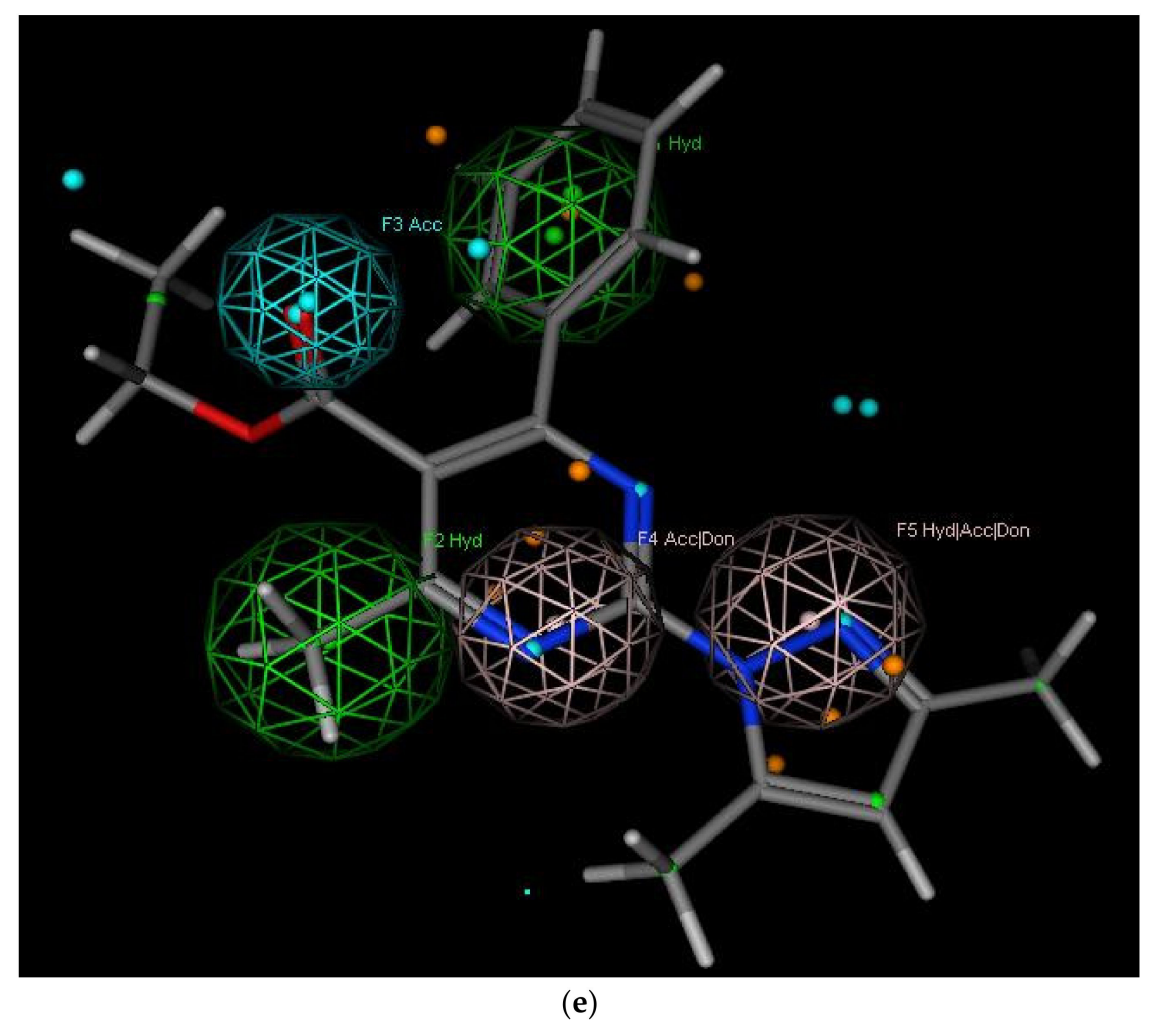
(**a**) Mapping of compound **2** on the pharmacophore model. (**b**) Mapping of compound **3a** on the pharmacophore model. (**c**) Mapping of compound **3b** on the pharmacophore model. (**d**) Mapping of compound **4** on the pharmacophore model. (**e**) Mapping of compound **11** on the pharmacophore model.

Results (Figure 5a–e) indicated that all compounds showing in vitro calcium channel blocking activity except compound **13** were able to satisfy pharmacophoric features of the model with RMSD values in the range 0.5678–0.8392, suggesting that they may share the same binding site on the receptor. Although compound **13** is active, it failed to fit the model, and this non-agreement might suggest a different binding mode. 

#### 2.3.2. *In Silico* Physicochemical Properties, Drug-Likeness and ADME

Recent drug discovery programs utilize *in silico* prediction of physicochemical and ADME parameters as useful lead identification tools. In this study, the physicochemical parameters formulating Lipinski’s rule [32] were computed for the most active compounds utilizing *Molinspiration* software [33] (Table 6). Interestingly, the selected compounds **2** and **11** were in full accordance with Lipinski’s parameters. *Molinspiration* [33] was also employed to calculate topological polar surface area (TPSA), which is utilized to calculate the estimated absorption percentage [34] as an additional bioavailability descriptor [35]. Herein, compounds **2** and **11** displayed drug-like TPSA values (<140–150 A^2^) [36,37] and reasonable absorption percentages (77–84%), predicting promising oral bioavailability. Aqueous solubility of **2** and **11** and their drug-likeness scores (Table 6) were predicted utilizing *Molsoft* software [38]. Both compounds recorded excellent drug-like predicted solubility and drug-likeness model scores.

Furthermore, *Pre-ADMET* software [39] was used for ADME prediction of the selected compounds. Accordingly, CaCo2 and MDCK cell permeability coefficients, human intestinal absorption (HIA), blood–brain barrier penetration (BBB), plasma protein binding (PPB) and inhibition of cytochromes P450 2D6 (CYP2D6) and P450 3A4 (CYP3A4) were computed and listed in Table 6. Both **2** and **11** displayed acceptable CaCo2 cell model permeability values (20.44 and 33.73 nm/s, respectively) and MDCK cell model permeability values (77.38 and 18.32 nm/s, respectively). Their HIA (92–98%) and BBB (0.67 and 1.58, respectively) values demonstrated excellent predicted intestinal absorption and acceptable CNS bioavailability of both compounds. Additionally, **2** was predicted to be devoid of the undesirable CYP3A4 and CYP2D6 inhibition activities, and hence potential drug–drug interactions are most probably excluded. 

### 2.4. Structure–Activity Relationship

**Figure 6:**
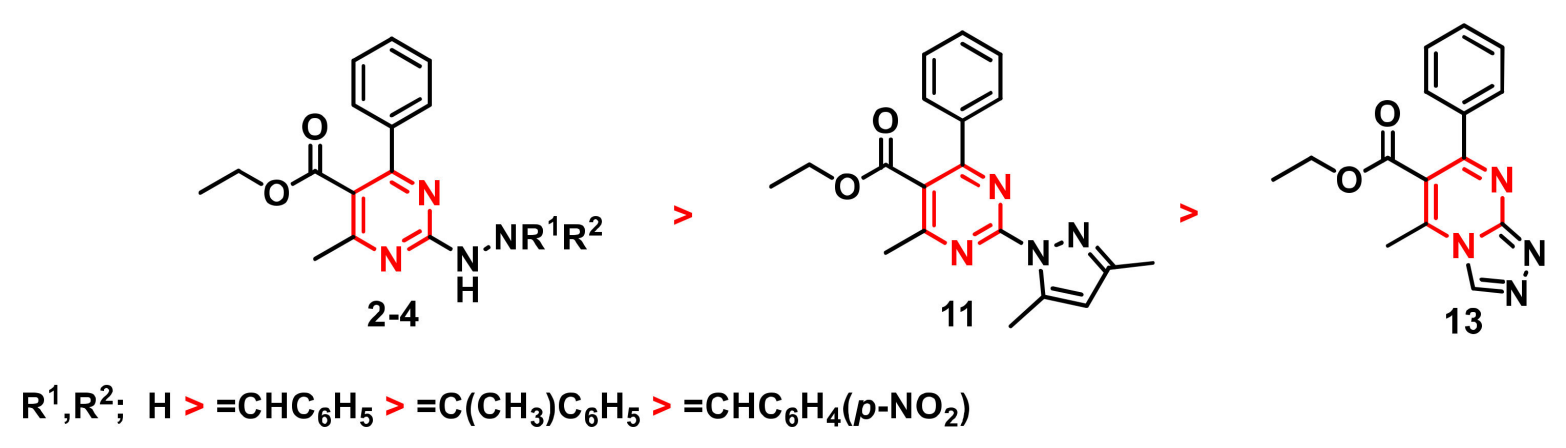
Summarized SAR pattern of the Biginelli-derived pyrimidine and fused pyrimidine CCBs.

The preliminary calcium channel blockade screening (Table 2) revealed that the designed 2-substituted Biginelli-derived scaffolds (Figure 2), when appropriately substituted, conserved the intrinsic calcium channel blocking activities of their DHP mimics [40], DHPM precursors [11,12,13,14,15,20] and pyrimidine-based leads [9]. It is worth mentioning this observation echoes previous structure–activity relation (SAR) studies showing that nifedipine [40] and DHPM CCBs [15,28,29,41] tolerate various C^2^ substituents. The promising group (Figure 6) included the 2-hydrazino derivative **2**, its hydrazones **3a**,**b** and **4**, the dimethyl-1*H*-pyrazol-1-yl derivative **11** and the triazolopyrimidine derivative **13**. Quantitative assessment of active compounds (Table 3) showed that most of the derivatives, namely **2**, **3a**, **3b** and **4**, that can display (donate) hydrogen bond(s) within the vicinity of the heterocyclic core showed notable calcium channel blockade activities. This correlation is consistent with previous SAR studies highlighting the critical hydrogen bonding interaction offered by the heterocyclic core of various DHP and DHPM CCBs. [42,43]. Herein, this hypothesis was supported by the elucidated pharmacophore model (Figure 4). However, it seems that the C^2^ substituent’s size and the number of possible hydrogen bond donors tuned the compounds’ potency (Table 3). The hydrazino moiety in compound **2** conferred the highest potency (IC_50_ = 0.96 µM) to the Biginelli-derived scaffold, followed by 2-benzylidenehydrazino (IC_50_ = 1.089 µM) and 2-(phenylethylidene)hydrazino (IC_50_ = 1.889 µM) groups in thee hydrazones **3a** and **4**, respectively. Notably, the introduction of the *p-*nitro group to the 2-benzylidenehydrazino motif in **3b** critically decreased the potency (IC_50_ = 2.82 µM) by approximately 2.5-fold relative to the unsubstituted derivative **3a**. Obviously, thiocarbamoylation, sulfonation, acylation and condensation with ethyl acetoacetate afforded the inactive derivatives **5a**,**b**, **8a**,**b**, **9** and **10**, respectively. These results point to the unfavorable effect of introducing electron-withdrawing and/or bulky groups to the hydrazino group on calcium channel antagonism. Another correlation between *C^2^* flexibility and the calcium channel blockade could be deduced from monitoring the activity of the pyrazolyl **11** and the triazolopyrimidine **13** derivatives, where the free hydrazino group was encaged in isolated or fused ring systems, respectively. Results (Table 3) showed that the pyrazolyl derivative **11** (IC_50_ = 2.594 µM) was 2.7-fold less potent than the hydrazino derivative **2**, whereas the triazolopyrimidine derivative **13** was the least potent (IC_50_ = 3.199 µM) among the group (3-fold less potent than **2**). These findings clarified the influence of C^2^ substituent flexibility on calcium channel antagonism. Again, introducing electron-withdrawing moieties to the ring systems was detrimental to activity, as evidenced by loss of activity in the case of the dioxopyrazolidin-1-yl derivative **12** and the pyrimidotriazine derivatives **14a**,**b**. Collectively, it could be concluded that the designed Biginelli-derived scaffold was optimized as the 2-hydrazino derivative **2**. It may tolerate hydrazones or isolated heterocycles of suitable size and electronic environment. On the other hand, it may be deduced that derivatizing the C^2^ hydrazino group into various electron-withdrawing functionalities either flexible (thiosemicarbzides **5a**,**b**, arylsulfonylhydrazines **8a**,**b**, ethoxycarbonylhydrazine **9** and ethoxyoxobutan-3-ylidene hydrazine **10**), in ring systems (thiazolidinone **6**, thiazoline **7** and pyrazolidinedione **12**) or fused with the heterocyclic core (pyrimidotriazines **14a**,**b**) was detrimental to activity.

In other words, the hydrazino group may be utilized as a spacer to introduce aromatic moieties taking into consideration keeping the linker flexible while avoiding electron-withdrawing groups. 

Further evaluation of selected CCBs (**2** and **11**) for their hypotensive activities (mg/kg, i.v.) in normotensive anesthetized dogs at different doses (Table 4) revealed that the pyrazolyl derivative **11** exhibited superior in vivo hypotensive activity relative to the hydrazino derivative **2**, at doses up to 12 mg/kg, despite being less active as a CCB according to in vitro studies. This poor correlation between the in vitro calcium channel blockade and in vivo hypotensive activities when prioritizing the evaluated derivatives refers to the influence of a secondary hypotensive mechanism that might have contributed to the in vivo potency of the pyrazolyl derivative **11.** Interestingly, higher doses (at 24 mg/kg i.v.) of both compounds exhibited approximately the same potency.

## 3. Materials and Methods

### 3.1. Chemistry

Melting points were determined in open-glass capillaries using a *Griffin* melting point apparatus and are uncorrected. IR spectra (KBr) were recorded using a *Bruker Vector 22* spectrophotometer at the Microanalytical Center, Faculty of Science, Cairo University. ^1^H NMR spectra were scanned on a *Mercury* spectrometer (300 MHz) at the Faculty of Science, Cairo University. ^13^C NMR, distortionless enhancement by polarization transfer (DEPT) and heteronuclear multiple bond coherence (HMBC) spectra were recorded on *Jeol* spectrometer (500 MHz) at the National Research Centre, Dokki, Cairo, using tetramethylsilane (TMS) as internal standard and DMSO-*d*_6_ as the solvent (chemical shifts are given in δ ppm). Splitting patterns were designated as follows: *s* = singlet, *d* = doublet, *t* = triplet, *q* = quartet, *m* = multiplet, *br* = broad, *dist* = distorted. Mass spectra were recorded using a *Shimadzu GCMS-Qp2010* plus (70 ev) at the Faculty of Science, Cairo University. Microanalyses were performed at the Microanalytical Unit, Faculty of Science, Cairo University. Results of the microanalyses were within ±0.4% of the calculated values. Follow-up of the reactions and checking the purity of the compounds were performed by thin-layer chromatography (TLC) on aluminum sheets precoated with silica gel (Type 60 GF254; Merck, Germany) and the spots were detected by exposure to UV lamp at 254 nm for a few seconds. Compound **1** was synthesized as described in [19]. 

Ethyl 2-hydrazino-6-methyl-4-phenylpyrimidine-5-carboxylate (**2**): Hydrazine hydrate 99% (3.75 g, 75 mmol) was slowly added to a solution of the methylsulfonyl derivative **1** (8 g, 25 mmol) in absolute EtOH (15 mL). The reaction mixture was stirred at RT for 30 min, during which complete dissolution and reprecipitation occurred. The obtained product was filtered, washed thoroughly with H_2_O, dried and crystallized from EtOH/H_2_O (5:1). Yield: (quantitative), m.p: 94–96 °C. IR (KBr, cm^−1^): 3254, 3221, 3060 (NH_2_, NH), 1710 (C=O), 1553 (C=N and C=C Ar), 1436 (C=C Ar), 1260, 1082 (υ_as_ and υ_s_ C-O-C). ^1^H-NMR (DMSO-*d*_6_, 300 MHz) δ ppm: 0.91 (*t*, *J* = 7.2 Hz, 3H, CH_3_CH_2_), 2.39 (*s*, 3H, C^6^-CH_3_), 4.01 (*q*, *J* = 7.2 Hz, 2H, CH_3_CH_2_), 4.33 (*s*, *br*, 2H, NH_2_, D_2_O-exchangeable), 7.40–7.55 (*m*, 5H, Ar-Hs), 8.62 (*s*, 1H, NH, D_2_O-exchangeable). EI-MS *m*/*z* (relative intensity): 272 ([M^+^], 100). Anal. Calcd. for C_14_H_16_N_4_O_2_ (272.3): C 61.75, H 5.92, N 20.58. Found: C 62.02, H 5.88, N 21.00. 

Ethyl 2-(2-arylidenehydrazino)-6-methyl-4-phenylpyrimidine-5-carboxylates (**3a**,**b**): A solution of hydrazine derivative **2** (0.27 g, 1 mmol) in absolute EtOH (5 mL) was treated with a solution of an equimolar amount of the appropriate aromatic aldehyde in absolute EtOH. The reaction mixture was heated under reflux for 15 h and cooled. The separated product was filtered, washed with petroleum ether (40–60°), dried and crystallized from EtOH. 

Ethyl 2-(2-benzylidenehydrazino)-6-methyl-4-phenylpyrimidine-5-carboxylate (**3a**): Yield: 63%, m.p: 133–135 °C. IR (KBr, cm^−1^): 3199 (NH), 1719 (C=O), 1578, 1541 (C=N occasionally mixed with C=C Ar), 1443 (C=C Ar), 1268, 1081 (υ_as_ and υ_s_ C-O-C). ^1^H-NMR (DMSO-*d*_6_, 300 MHz) δ ppm: 0.94 (*t*, *J* = 7.2 Hz, 3H, CH_3_CH_2_), 2.47 (*s*, 3H, C^6^-CH_3_), 4.06 (*q*, *J* = 7.2 Hz, 2H, CH_3_CH_2_), 7.34–7.71 (*m*, 10H, Ar-Hs), 8.20 (*s*, 1H, N=CH), 11.64 (*s*, 1H, NH, D_2_O-exchangeable). Anal. Calcd. for C_21_H_20_N_4_O_2_ (360.41): C 69.98, H 5.59, N 15.55. Found: C 70.08, H 6.00, N 15.50. 

Ethyl 6-methyl-2-(2-(4-nitrobenzylidene)hydrazino)-4-phenylpyrimidine-5-carboxylate (**3b**): Yield: 69%, m.p: 220–222 °C. IR (KBr, Cm-1): 3325 (NH), 1706 (C=O), 1544 (C=N occasionally mixed with C=C Ar), 1434 (C=C Ar), 1257, 1082 (υ_as_ and υ_s_ C-O-C). ^1^H-NMR (DMSO-*d*_6_, 300 MHz) δ ppm: 0.96 (*t*, *dist*, *J* = 7.2 Hz, 3H, CH_3_CH_2_), 2.50 (*s*, 3H, C^6^-CH_3_), 4.09 (*q*, *dist*, *J* = 7.5 Hz, 2H, CH_3_CH_2_), 7.50–7.58 (*m*, 5H, Ar-Hs), 7.93 (*d*, *dist*, *J* = 8.7 Hz, 2H, Ha, C^2′,6′^-Hs), 8.25 (*s*, 1H, N=CH), 8.27 (*d*, *J* = 9 Hz, 2H, Hb, C^3′,5′^-Hs), 11.95 (*s*, 1H, NH, D_2_O-exchangeable). EI-MS *m*/*z* (relative intensity): 405 ([M^+^], 14), 228 (100). Anal. Calcd. for C_21_H_19_N_5_O_4_ (405.41): C 62.22, H 4.72, N 17.27. Found: C 62.09, H 4.83, N 16.91. 

Ethyl 2-[2-(1-phenylethylidene)hydrazino]-6-methyl-4-phenylpyrimidine-5-carboxylate (**4**): A solution of hydrazine **2** (0.27 g, 1 mmol) in absolute EtOH (5 mL) was treated with a solution of an equimolar amount of acetophenone in absolute EtOH. The reaction mixture was heated under reflux for 19 h and cooled to RT. The separated product was filtered, washed with petroleum ether (40–60°), dried and crystallized from EtOH unless otherwise stated. Yield: 75%, m.p: 162–164 °C. IR (KBr, cm^−1^): 3223 (NH), 1717 (C=O), 1551 (C=N occasionally mixed with C=C Ar), 1438 (C=C Ar), 1259, 1082 (υ_as_ and υ_s_ C-O-C). ^1^H-NMR (DMSO-*d*_6_, 300 MHz) δ ppm: 0.94 (*t*, *J* = 7.2 Hz, 3H, CH_3_CH_2_), 2.35 (*s*, 3H, N=C-CH_3_(Ar)), 2.49 (*s*, 3H, C^6^-CH_3_ overlapping with DMSO), 4.06 (*q*, *J* = 7.5 Hz, 2H, CH_3_CH_2_), 7.36–7.83 (*m*, 10H, Ar-Hs), 10.50 (*s*, 1H, NH, D_2_O-exchangeable). ^13^C-NMR (DMSO-*d*_6_, 125 MHz) δ ppm: 14.0, 14.4, 23.1, 61.5, 117.5, 126.6, 128.4, 128.9, 129.2, 130.2, 138.8, 139.1, 149.4, 160.0, 165.7, 167.1, 168.4; EI-MS *m*/*z* (relative intensity): 374 ([M^+^], 64), 373 (100). Anal. Calcd. for C_22_H_22_N_4_O_2_ (374.44): C 70.57, H 5.92, N 14.96. Found: C 70.79, H 6.02, N 15.05.

Ethyl 6-methyl-4-phenyl-2-[2-(substituted thiocarbamoyl)hydrazino]pyrimidine-5-carboxylates (**5a**,**b**): The appropriate isothiocyanate derivative (0.01 mol) was added to a well-stirred suspension of hydrazine derivative **2** (2.7 g, 0.01 mol) in absolute EtOH (20 mL). The reaction mixture was stirred at RT for 2 h, during which dissolution and reprecipitation occurred. The obtained precipitate was filtered, washed with petroleum ether (40–60°), dried and crystallized from EtOH. 

Ethyl 6-methyl-4-phenyl-2-[2-(phenylthiocarbamoyl)hydrazino]pyrimidine-5-carboxylates (**5a**): Yield: 90%, mp: 140–142 °C. IR (KBr, cm^−1^): 3274, 3151 (NH), 1715 (C=O), 1557 (C=N mixed with C=C Ar), 1438 (C=C Ar), 1526, 1358, 1219, 1016 (N-C=S amide I, II, III, IV bands), 1252, 1089 (υ_as_ and υ_s_ C-O-C).^1^H-NMR (DMSO-*d*_6_, 300 MHz) δ ppm: 0.97 (*t*, *J* = 7.2 Hz, 3H, CH_3_CH_2_), 2.44 (*s*, 3H, C^6^-CH_3_), 4.08 (*q*, *J* = 7.2 Hz, 2H, CH_3_CH_2_), 7.13–7.56 (*m*, 10H, Ar-Hs), 9.52 (*s*, 1H, NHNHC=S, D_2_O-exchangeable), 9.73 (*s*, 1H, NHC_6_H_5_, D_2_O-exchangeable), 9.75 (*s*, 1H, C^2^-NH, D_2_O-exchangeable). ^13^C-NMR (DMSO-*d*_6_, 125 MHz) δ ppm: 14.0, 23.0, 61.7, 117.7, 125.4, 126.1, 128.4, 128.9, 130.4, 138.5, 139.9, 162.2, 165.0, 166.9, 168.5, 181.6; Anal. Calcd. for C_21_H_21_N_5_O_2_S.H_2_O (425.50): C 59.28, H 5.45, N 16.46. Found: C 59.47, H 5.05, N 15.75.

Ethyl 2-[2-(butyl thiocarbamoyl)hydrazino]-6-methyl-4-phenylpyrimidine-5-carboxylates (**5b**): Yield: 97%, m.p: 125–127 °C. IR (KBr, cm^−1^): 3226 (NH), 1724 (C=O), 1565 (C=N mixed with C=C Ar), 1439 (C=C Ar), 1539, 1377, 1171, 1020 (N-C=S amide I, II, III, IV bands), 1253, 1088 (υ_as_ and υ_s_ C-O-C). ^1^H-NMR (DMSO-*d*_6_, 300 MHz) δ ppm: 0.83 (*t*, *J* = 7.2 Hz, 3H, C^d^H_3_), 0.96 (*t*, *J* = 7.2 Hz, 3H, ester CH_3_), 1.24 (*m*, 2H, C^c^H_2_), 1.47 (*m*, 2H, C^b^H_2_), 2.41 (*s*, 3H, C^6^-CH_3_), 3.44 (*q*, *J* = 6.6 Hz, 2H, C^a^H_2_, appearing as *t* after deuteration), 4.08 (*q*, *J* = 7.2 Hz, 2H, ester CH_2_), 7.40–7.46 (*m*, 5H, C^4^-Ar-Hs), 8.02 (*t*, *J* = 6.9 Hz, br, 1H, NHC_4_H_9_, D_2_O-exchangeable), 9.23 (*s*, 1H, NHC=S, D_2_O-exchangeable), 9.30 (*s*, 1H, C^2^-NH, D_2_O-exchangeable). Anal. Calcd. for C_19_H_25_N_5_O_2_S (387.5): C 58.89, H 6.50, N 18.07. Found: C 58.77, H 6.37, N 18.69.

Ethyl 6-methyl-2-[2-(3-butyl-4-oxothiazolidin-2-ylidene)-hydrazino]-4 phenylpyrimidine-5-carboxylate (**6**): Ethyl bromoacetate (0.167 g, 1 mmol) was added to a suspension of thiosemicarbazide derivative **5b** (1 mmol) in absolute EtOH (5 mL) containing anhydrous NaOAc (0.12 g, 1.5 mmol). The reaction mixture was heated under reflux for 3 h, concentrated to half its volume, allowed to attain RT and poured onto ice-cold H_2_O (10 mL). The obtained precipitate was filtered, dried and crystallized from EtOH. Yield: 62%, m.p: 121–123 °C. IR (KBr, cm^−1^): 3159 (NH), 1710 (C=O ester), 1624 (C=O amide), 1553 (C=N), 1470 (C=C Ar), 1262, 1092 (υ_as_ and υ_s_ C-O-C), 1168, 1018 (υ_as_ and υ_s_ C-S-C). **^13^C-NMR** (DMSO-*d*_6_, 125 MHz) δ ppm: 13.9, 14.2, 20.0, 23.1, 29.2, 33.0, 42.8, 61.4, 116.4, 128.4, 128.8, 130.2, 138.9, 159.4, 161.1, 165.3, 166.9, 168.5, 172.1; Anal. Calcd. for C_21_H_25_N_5_O_3_S (427.54): C 59.00, H 5.89, N 16.38. Found: C 59.13, H 5.94, N 16.47

Ethyl 2-[2-(4-(4-bromophenyl)-3-phenylthiazol-2(3H)-ylidene)hydrazino]-6-methyl-4-phenylpyrimidine-5-carboxylate (**7**): A solution of 4-bromophenacyl bromide (1 mmol) in absolute EtOH (5 mL) was gradually added to a suspension of the thiosemicarbazide derivative 5a (1 mmol) and the equimolar amount of anhydrous NaOAc (0.082 g, 1 mmol) in absolute EtOH (5 mL). The reaction mixture was heated under reflux for 3–4 h, concentrated to half its volume and left to attain RT. The obtained precipitate was filtered, washed with H_2_O and crystallized from EtOH. Yield: 54%, m.p: 172–174 °C. IR (KBr, cm^−1^): 3161 (NH), 1713 (C=O), 1627 (C=N), 1584, 1497 (C=C Ar), 1259, 1087 (υ_as_ and υ_s_ C-O-C), 1150, 1039 (υ_as_ and υ_s_ C-S-C). **^1^H-NMR** (DMSO-*d*_6_, 300 MHz) δ ppm: 0.97 (*t*, *J* = 7.2 Hz, 3H, CH_3_CH_2_), 2.41 (*s*, 3H, C^6^-CH_3_), 4.08 (*q*, *J* = 7.2 Hz, 2H, CH_3_CH_2_), 6.47 (*s*, 1H, thiazoline-*H*), 6.82–7.55 (*m*, 12 H, Ar-Hs), 7.64 (*d*, *J* = 8.4 Hz, 2H, Hb), 10.38, 10.43 (2 *s*, 1H, NH, D_2_O exchangeable). Anal. Calcd. for C_29_H_24_BrN_5_O_2_S (586.5): C 59.39, H 4.12, N 11.94. Found: C 59.15, H 3.87, N 11.83. 

2-[2-(Arylsulfonyl)hydrazino]-6-methyl-4-phenylpyrimidine-5-carboxylates (**8a**,**b**): A mixture of hydrazine **2** (0.27 g, 1 mmol) and the appropriate arylsulfonyl chloride (1 mmol) in dry pyridine (5 mL) was stirred at RT for 24 h. The reaction mixture was diluted with crushed ice and neutralized with dilute HCl (10%). The obtained precipitate was filtered, dried and crystallized from benzene. 

Ethyl 6-methyl-4-phenyl-2-[2-(phenylsulfonyl)hydrazino]-pyrimidine-5-carboxylate (**8a**): Yield: 66%, m.p: 98–100 °C. IR (KBr, Cm-1): 3218 (NH), 1719 (C=O), 1556 (C=N mixed with C=C Ar), 1438 (C=C Ar), 1342, 1170 (υ_as_ and υ_s_ SO_2_), 1268, 1089 (υ_as_ and υ_s_ C-O-C). ^1^H-NMR (DMSO-*d*_6_, 300 MHz) δ ppm: 0.93 (*t*, *J* = 7.5 Hz, 3H, CH_3_CH_2_), 2.22 (*s*, *br*, 3H, C^6^-CH_3_), 4.03 (*q*, *J* = 7.2 Hz, 2H, CH_3_CH_2_), 7.28–7.80 (*m*, 10H, Ar-Hs), 9.71 (*s*, 1H, C^2^-NH, D_2_O-exchangeable), 9.85 (*s*, 1H, NHSO_2_, D_2_O-exchangeable). Anal. Calcd. for C_20_H_20_N_4_O_4_S (412.46): C 58.24, H 4.89, N 13.58. Found: C 58.51, H 5.20, N 15.32.

Ethyl 6-methyl-4-phenyl-2-[2-(4-tolylsulfonyl)hydrazino]-pyrimidine-5-carboxylate (**8b**): Yield: 64%, m.p: 127–129 °C. IR (KBr, cm^−1^): 3211 (NH), 1721 (C=O), 1555 (C=N mixed with C=C Ar), 1438 (C=C Ar), 1345, 1165 (υ_as_ and υ_s_ SO_2_), 1266, 1088 (υ_as_ and υ_s_ C-O-C).^1^H-NMR (DMSO-*d*_6_, 300 MHz) δ ppm: 0.92 (*t*, *J* = 7.5 Hz, 3H, CH_3_CH_2_), 2.22 (*s*, *br*, 6H, C^6^-CH_3_ + C^4′’^-CH_3_), 4.02 (*q*, *J* = 7.5 Hz, 2H, CH_3_CH_2_), 7.33 (*d*, *J* = 7.8 Hz, *dist*, 2H, Ha), 7.40–7.51 (*m*, 5H, Ar-Hs), 7.60 (*d*, *J* = 7.8 Hz, 2H, Hb), 9.71 (*s*, 1H, C^2^-NH, D_2_O-exchangeable), 9.80 (*s*, 1H, NHSO_2_, D_2_O-exchangeable). ^13^C-NMR (DMSO-*d*_6_, 125 MHz) δ ppm: 13.9, 21.4, 22.6, 61.6, 116.9, 127.9, 128.2, 128.6, 129.5, 130.3, 137.2, 138.2, 143.4, 161.3, 164.6, 166.7, 168.3; EI-MS *m*/*z* (relative intensity): 426 ([M^+^], 20), 242 (100). Anal. Calcd. for C_21_H_22_N_4_O_4_S (426.49): C 59.14, H 5.20, N 13.14. Found: C 59.49, H 5.00, N 13.00.

Ethyl 2-[2-(ethoxycarbonyl)hydrazino]-6-methyl-4-phenylpyrimidine-5-carboxylate (**9**): Ethyl chloroformate (0.165 g, 1.5 mmol) was added to a suspension of the hydrazine **2** (0.27 g, 1 mmol) and anhydrous K_2_CO_3_ (0.27 g, 2 mmol) in dry dioxane (5 mL). The reaction mixture was heated under reflux while stirring for 5 h, left to cool to RT, diluted with ice-cold H_2_O (30 mL) and refrigerated overnight. The separated product was filtered, dried and crystallized from CH_2_Cl_2/_petroleum ether (40–60°) (1:4). Yield: 70%, m.p: 112–114 °C. IR (KBr, cm^−1^): 3332, 3307 (NH), 1710 (C=O), 1557 (C=N mixed with C=C Ar), 1444 (C=C Ar), 1256, 1089 (υ_as_ and υ_s_ C-O-C).^1^H-NMR (DMSO-*d*_6_, 300 MHz) δ ppm: 0.97 (*t*, *J* = 7.2 Hz, 3H, C^5^-ester CH_2_CH_3_), 1.21 (*t*, *J* = 7.2 Hz, 3H, carbamate CH_2_CH_3_), 2.49 (*s*, 3H, C^6^-CH_3_), 4.07 (2 overlapping *q*, *J* = 7.2 Hz, 4H, 2 × CH_2_), 7.40–7.55 (*m*, 5H, Ar-Hs), 8.76 and 9.13 (2*s*, *br*, 1H, C^2^-NH, D_2_O-exchangeable), 9.33 (*d*, *dist*, 1H, NHCOOEt, D_2_O-exchangeable). EI-MS *m*/*z* (relative intensity): 344 ([M^+^], 14), 242 (100). Anal. Calcd. for C_17_H_20_N_4_O_4_ (344.37): C 59.29, H 5.85, N 16.27. Found: C 59.00, H 5.60, N 16.50.

Ethyl 2-[2-(1-ethoxy-1-oxobutan-3-ylidene)hydrazino]-6-methyl-4- phenylpyrimidine-5-carboxylate (**10**): A mixture of hydrazine derivative **2** (0.27 g, 1 mmol) and ethyl acetoacetate (0.13 g, 1 mmol) in absolute EtOH (5 mL) was heated under reflux for 4 h. the reaction mixture was concentrated and cooled to RT. The obtained precipitate was filtered, washed with petroleum ether (40–60°), dried and crystallized from EtOH. Yield: 64%, m.p: 98–100 °C. IR (KBr, cm^−1^): 3196 (NH), 1721 (C=O), 1577 (C=N mixed with C=C Ar), 1544 (C=C Ar), 1255, 1087 (υ_as_ and υ_s_ C-O-C).^1^H-NMR (DMSO-*d*_6_, 300 MHz) δ ppm: 0.93 (*t*, *J* = 7.2 Hz, 3H, C^5^-CO_2_CH_2_CH_3_), 1.20 (*t*, *J* = 7.2 Hz, 3H, CH_2_CO_2_CH_2_CH_3_), 2.00 (*s*, 3H, CH_3_-C=N), 2.44 (*s*, 3H, C^6^-CH_3_), 3.36 (*s*, 2H, CH_2_CO_2_C_2_H_5_), 4.05 (*q*, *J* = 7.2 Hz, 2H, C^5^-CO_2_CH_2_CH_3_), 4.11 (*q*, *J* = 7.2 Hz, 2H, CH_2_CO_2_CH_2_CH_3_), 7.49–7.51 (*m*, 5H, Ar-Hs), 10.17 (*s*, 1H, NH, D_2_O-exchangeable). ^13^C-NMR (DMSO-*d*_6_, 125 MHz) δ ppm: 13.9, 14.6, 17.1, 22.9, 40.2, 61.0, 61.5, 117.3, 128.3, 128.8, 130.2, 138.8, 148.5, 159.9, 165.7, 167.0, 168.3, 170.5; EI-MS *m*/*z* (relative intensity): 384 ([M^+^], 6), 297 (100). Anal. Calcd. for C_20_H_24_N_4_O_4_ (384.43): C 62.49 H 6.29, N 14.57. Found: C 62.56, H 6.38, N 15.00. 

Ethyl 6-methyl-2-(3,5-dimethyl-1H-pyrazol-1-yl)-4-phenylpyrimidine-5-carboxylate (**11**): A mixture of hydrazine derivative **2** (0.27 g, 1 mmol) and acetylacetone (0.1 g, 1 mmol) in absolute EtOH (5 mL) was heated under reflux for 5 h. The reaction mixture was concentrated, diluted with ice-cold H_2_O (20 mL) and refrigerated overnight. The obtained precipitate was filtered, dried and crystallized from petroleum ether (40–60°). Yield: 92%, m.p: 60–62 °C. IR υ (KBr, cm^−1^): 1719 (C=O), 1550 (C=N mixed with C=C Ar), 1437 (C=C Ar), 1263, 1093 (υ_as_ and υ_s_ C-O-C). ^1^H-NMR (DMSO-*d*_6_, 300 MHz) δ ppm: 1.04 (*t*, *J* = 7.2 Hz, 3H, CH_3_CH_2_), 2.21 (*s*, 3H, C^6^-CH_3_), 2.59 (*s*, 3H, C^3″^-CH_3_), 2.62 (*s*, 3H, C^5″^-CH_3_), 4.20 (*q*, *J* = 7.2 Hz, 2H, CH_3_CH_2_), 6.19 (*s*, 1H, C^4′’^-H), 7.51–7.67 (*m*, 5H, Ar-Hs). ^13^C-NMR (DMSO-*d*_6_, 125 MHz) δ ppm: 14.0, 15.5, 23.0, 62.3, 110.8, 122.4, 128.7, 129.2, 131.0, 137.4, 143.2, 150.9, 156.2, 164.8, 167.6, 167.8; EI-MS *m*/*z* (relative intensity): 336 ([M^+^], 100). Anal. Calcd. for C_19_H_20_N_4_O_2_ (336.39): C 67.84, H 5.99, N 16.66. Found: C 67.79, H 6.13, N 16.54

Ethyl 6-methyl-2-(3,5-dioxopyrazolidin-1-yl)-4-phenylpyrimidine-5-carboxylate (**12**): A mixture of hydrazine **2** (0.27 g, 1 mmol) and diethyl malonate (0.32 g, 2 mmol) in absolute EtOH/glacial HOAc (4:1) (5 mL) was heated under reflux for 11 h, concentrated to a small volume and diluted with ice-cold H_2_O. The separated product was filtered, dried and crystallized from CH_2_Cl_2/_petroleum ether (40–60°) (1:4). Yield: 42%, m.p: 166–168 °C. IR (KBr, cm^−1^): 3327, 3276 (OH, NH), 1710 (C=O ester), 1668 (C=O amide), 1557 (C=N mixed with C=C Ar), 1440 (C=C Ar), 1263, 1091 (υ_as_ and υ_s_ C-O-C). ^1^H-NMR (DMSO-*d*_6_, 300 MHz) δ ppm: 0.90 (*t*, *J* = 6.85 Hz, 3H, CH_2_CH_3_), 1.87 (*s*, 3H, C^6^-CH_3_), 4.01 (*q*, *J* = 6.75 Hz, 2H, CH_2_CH_3_), 4.13 (*s*, 1H, C^4′’^-H), 7.44 (*s*, 5H, Ar-Hs), 9.25 (*s*, 1H, NH, D_2_O-exchangeable), 9.83 (*s*, *br*, 1H, OH, D_2_O-exchangeable). ^13^C-NMR (DMSO-*d*_6_, 125 MHz) δ ppm: 13.91, 21.1, 61.5, 116.8, 128.3, 128.9, 130.3, 138.7, 162.1, 162.5, 165.2, 167.0, 168.5, 169.7; Anal. Calcd. for C_17_H_16_N_4_O_4_ (340.33): C 59.99, H 4.74, N 16.46. Found: C 59.54, H 5.10, N 16.68. 

Ethyl 5-methyl-7-phenyl-1,2,4-triazolo[4,3-a]pyrimidine-6-carbxylate (**13**): A solution of hydrazine derivative **2** (0.27 g, 1 mmol) in formic acid (5 mL) was heated under reflux for 27 h. The reaction mixture was concentrated to a small volume and diluted with ice-cold H_2_O. The obtained precipitate was filtered, washed with H_2_O, dried and crystallized from EtOH/H_2_O. Yield 34%, m.p: 104–106 °C. IR (KBr, cm^−1^): 1724 (C=O), 1608 (C=N), 1520, 1442 (C=C Ar), 1285, 1087 (υ_as_ and υ_s_ C-O-C). ^1^H-NMR (DMSO-*d*_6_, 300 MHz) δ ppm: 0.96 (*t*, *J* = 7.5 Hz, 3H, CH_3_CH_2_), 2.90 (*s*, 3H, C^5^-CH_3_), 4.15 (*q*, *J* = 7.5 Hz, 2H, CH_3_CH_2_), 7.50–7.63 (*m*, 5H, Ar-Hs), 8.80 (*s*, 1H, C^3^-H). ^13^C-NMR (DMSO-*d*_6_, 125 MHz) δ ppm: 13.4, 15.4, 62.2, 116.9, 128.1, 128.7, 130.2, 138.0, 148.5, 153.7, 157.0, 160.9, 165.5; EI-MS *m*/*z* (relative intensity): 282 ([M^+^], 54), 253 (100). Anal. Calcd. for C_15_H_14_N_4_O_2_ (282.3): C 63.82, H 5.00. N 19.85. Found: C 63.86, H 5.05, N 19.00

Ethyl 6-methyl-8-phenyl-3-(substituted phenyl)-4H-pyrimido[2,1-c]-1,2,4-triazine-7-carboxylates (**14a**,**b**): The appropriate 4-substituted phenacyl bromide (1 mmol) was added to a solution of the hydrazine derivative **2** (0.27 g, 1 mmol) in absolute EtOH (5 mL). The reaction mixture was heated under reflux for 2 h, concentrated to half its volume and then left to cool to RT. The obtained precipitate was filtered, washed with petroleum ether (40–60°), dried and crystallized from EtOH. 

Ethyl 6-methyl-3,8-diphenyl-4H-pyrimido[2,1-c]-1,2,4-triazine-7-carboxylate (**14a**): Yield: 22%, m.p: 234–236 °C (charring). IR (KBr, cm^−1^): 1724 (C=O), 1611 (C=N), 1553, 1446 (C=C Ar), 1257, 1049 (υ_as_ and υ_s_ C-O-C). **^1^H-NMR** (DMSO-*d*_6_, 300 MHz) δ ppm: 1.00 (*t*, *J* = 7.2 Hz, 3H, CH_3_CH_2_), 2.80 (*s*, 3H, C^6^-CH_3_), 4.20 (*q*, *J* = 7.2 Hz, 2H, CH_3_CH_2_), 5.56 (*s*, 2H, C^4^-H_2_), 7.57–7.96 (*m*, 10 H, Ar-Hs), 13.26 (*s*, 1H, NH, D_2_O-exchangeable). EI-MS *m*/*z* (relative intensity): 372 ([M^+^], 65), 77 (100). Anal. Calcd. for C_22_H_20_N_4_O_2_ (372.42): C 70.95, H 5.41, N 15.04. Found: C 58.86, H 5.02, N 14.89.

Ethyl 3-(4-bromophenyl)-6-methyl-8-phenyl-4H-pyrimido[2,1-c]-1,2,4-triazine-7-carboxylate (**14b**): Yield: 27%, m.p: 242–244 °C (charring). IR (KBr, cm^−1^): 1725 (C=O), 1615 (C=N), 1548, 1446(C=C Ar), 1256, 1056 (υ_as_ and υ_s_ C-O-C). ^1^H-NMR (DMSO-*d*_6_, 500 MHz) δ ppm: 0.97 (*t*, *J* = 6.9 Hz, 3H, CH_3_CH_2_), 2.79 (*s*, 3H, C^6^-CH_3_), 4.17 (*q*, *J* = 6.9 Hz, 2H, CH_3_CH_2_), 5.54 (*s*, 2H, C^4^-H_2_), 7.54–7.64 (m, 3H, Ar-Hs), 7.64–7.70 (m, 2H, Ar-Hs), 7.74 (*d*, *J* = 8.4Hz, 2H, Ha), 7.88 (*d*, *J* = 8.4Hz, 2H, Hb), 13.28 (*s*, 1H, NH, D_2_O-exchangeable). ^13^C-NMR (DMSO-*d*_6_, 125 MHz) δ ppm: 13.3, 17.5, 39.7, 62.9, 120.1, 125.7, 128.5, 128.6, 129.7, 131.6, 132.4, 133.0, 135.6, 144.7, 147.7, 160.6, 164.9, 169.6; Anal. Calcd. for C_22_H_19_BrN_4_O_2_ (451.32): C 58.55, H 4.24, N 12.41. Found: C 53.11, H 4.29, N 12.20.

### 3.2. Biological Evaluation

#### 3.2.1. Experimental Animals

Animals were obtained and housed in Moassat Hospital Animal House, Pharmacology Department, Faculty of Medicine, Alexandria University. Rats and rabbits were kept in cages with wide mesh wire bottoms under standard conditions of light and temperature and allowed food and H_2_O *ad libitum* (dogs were kept at separate theatres). The experimental protocol was approved by the Animal Care and Use Committee, Faculty of Pharmacy, Alexandria University (ACUC project number 15). 

#### 3.2.2. Data Recoding

Intestinal responses were recorded using an isometric transducer (Model TRI 201, Panlab S.I.) connected to an amplifier (Model Iso 510, Panlab S.I.). Tissues were mounted in a Bioscience organ bath.

In normotensive anesthetized dog experiments, mean arterial blood pressure (MAP) was recorded on a Grass polygraph via a pressure transducer (Model TRA 021, Panlab S.I.) triggered by an amplifier (Model Iso 510, Panlab S.I.) and connected to a mercury manometer.

#### 3.2.3. Statistical Analysis and Data Interpretation

Statistical analysis was conducted using GraphPad Prism version 3.02 software package [44] to calculate IC_50_, mean, standard deviation and standard error of each mean and for comparison between different groups involved. One-way test was used for comparison between independent samples.

#### 3.2.4. In Vitro Calcium Channel Blocking Activity 

##### Rat Colon

Thirty Wistar albino rats (200–250 g) of either sex were starved with free access to H_2_O for 24 h prior to experiments and sacrificed by cervical dislocation on the day of the experiment; the abdominal cavity was opened and the ascending colon was rapidly removed and immersed in Kreb’s solution of the following composition (mM): NaCl 118.4, KCl 4.7, MgSO_4_.H_2_O 1.2, KH_2_PO_4_.2H_2_O 1.2, NaHCO_3_ 25, CaCl_2_ 1.25 and glucose 11.1. 

Segments (1.5–2 cm) were mounted vertically under 1g tension in a 25 mL organ bath containing Kreb’s solution maintained at 37 °C and aerated with carbogen (95% O_2_ and 5% CO_2_). Preparations were allowed to equilibrate for about 30 min with regular washes. 

Solutions of nifedipine and test compounds in DMSO, selected for in vitro calcium channel blocking activity [26], were freshly prepared, protected from light and added to the organ bath to give a final concentration of 10^−5^ M. 

Tissues were contracted with 100 mM KCl and the maximum response was recorded. Tissues were then washed thoroughly with Kreb’s solution and, after reaching a steady state, were preincubated for 5 min with test compounds (10^−5^ M); again, KCl was added with the same final concentration and maximum contractions were recorded.

##### Rabbit Jejunum

Eight white New Zealand rabbits (1.5–2 kg) of either sex were starved with free access to H_2_O for 24 h prior to experiments and then slaughtered; the abdomen was opened and the jejunal portion was immediately isolated and kept in Tyrode’s solution of the following composition (mM): KCl 2.68, NaCl 136.9, MgCl_2_ 1.05, NaHCO_3_ 11.90, NaH_2_PO_4_ 0.42, CaCl_2_ 1.8 and glucose 5.55. 

Segments (1.5–2 cm) were mounted vertically under 1g tension in a 25 mL organ bath containing Tyrode’s solution maintained at 37 °C and aerated with carbogen (95% O_2_ and 5% CO_2_). Preparations were allowed to equilibrate for about 30 min with regular washes. The same steps were followed as in rat colon for preliminary screening. 

For quantitative studies, contractions produced by KCl (100 mM) were recorded in the absence and presence of different concentrations of active compounds. The percentage of inhibition of KCl-induced contractions was plotted against the concentration of the compounds for the determination of IC_50_. 

#### 3.2.5. In Vivo Hypotensive Activity on Normotensive Anesthetized Dogs 

Eight adult normotensive dogs (15–25 kg) of either sex were anesthetized with thiopental sodium (35 mg/kg, i.v.), and additional doses were administered when needed. A 5 cm incision was made in the skin of the groin and underlying muscles were cut. Both femoral vein and artery were exposed, and each was cannulated for drug administration and determination of arterial blood pressure, respectively. The arterial cannula was connected to the pressure transducer, and arterial blood pressure was then recorded on the manometer and changes were displayed on the polygraph. Normal saline (0.90% *w*/*v* NaCl) was infused slowly throughout the experiments.

Solutions of nifedipine and test compounds (0.7 M) in DMSO were injected i.v. [27], DMSO alone did not influence the dogs’ mean MAP in control experiments. At least 15 min was allowed between challenge doses and appropriate vehicle controls. Records for test compounds were compared to the corresponding control values.

## 4. Conclusions

The Biginelli-derived pyrimidines and fused pyrimidines **2**, **3a**, **3b**, **4**, **11** and **13** showed the highest ex vivo calcium channel blocking activities. It was noticed that the potency among the promising compounds could be a function of the number and size of possible hydrogen bond donors/acceptors at C^2^. The substituent flexibility also critically contributed to the detected activity. Moreover, **2** and **11** revealed good hypotensive activities in dogs. A ligand-based pharmacophore model described the binding mode of the newly synthesized active compounds. This may also serve as a reliable basis for designing new active pyrimidine-based CCBs. Finally, the selected most active compounds **2** and **11** displayed drug-like *in silico* ADME parameters. 

## Figures and Tables

**Table 1:** Preliminary screening of calcium channel blocking activity of the tested compounds at a concentration of 10^−5^ M in DMSO on isolated rat colon and rabbit jejunum (*n* = 4) ^a^.

Cpd No.	Rat Colon	Rabbit Jejunum	Cpd No.	Rat Colon	Rabbit Jejunum
**2**	+	+	**8b**	-	-
**3a**	+	+	**9**	-	-
**3b**	+	+	**10**	-	-
**4**	+	+	**11**	+	+
**5a**	-	-	**12**	-	-
**5b**	-	-	**13**	+	+
**6**	-	-	**14a**	-	-
**7**	-	-	**14b**	-	-
**8a**	-	-	Nifedipine	+	+

^a^ refers to the number of observations used. (+) refers to compounds inhibiting KCl-induced contractions. (-) refers to inactive compounds.

**Table 2:** Quantitative assessment of active compounds expressed as % inhibition of KCl-induced contractions on isolated rabbit jejunum at different concentrations (*n* = 4) ^a^.

Compound No.	% Inhibition of KCl-Induced Contractions		
	2 × 10^−5^ M	4 × 10^−5^ M	6 × 10^−5^ M
**2**	44.45 ± 26.06	66.67 ± 33.33	100 ± 0
**3a**	25.00 ± 14.43	62.50 ± 23.94	72.33 ± 14.68
**3b**	17.00 ± 9.82	89.00 ± 11.00	100 ± 0
**4**	11.33 ± 9.81	33.45 ± 16.78	55.56 ± 24.22
**11**	15.55 ± 4.45	50.00 ± 30.00	70.00 ± 15.28
**13**	25.09 ± 16.03	55.09 ± 17.94	12.50 ± 7.98
Nifedipine	100 ± 0		

^a^ refers to the number of experiments.

**Table 3:** Quantitative assessment of active compounds expressed as IC_50_ and PIC_50_ on isolated rabbit jejunum. (*n* = 3–4) ^a^.

Compound No.	IC_50_ (µM)	pIC_50_
**2**	0.96	6.017
**3a**	1.089	5.962
**3b**	2.82	5.549
**4**	1.889	5.723
**11**	2.594	5.586
**13**	3.199	5.494
Nifedipine	6.279 (nM)	8.202

^a^ refers to the number of experiments used. pIC_50_ scale = −log IC_50_ (higher values indicate exponentially greater potency).

**Table 4:** Hypotensive activity of selected test compounds (mg/kg, i.v.) in normotensive anesthetized dogs represented by change in MAP (mmHg) as mean ± SE (*n* = 3–5) ^a^.

Compound No.	Decrease in MAP (mmHg) as Mean ± SE		
	6 mg/kg	12 mg/kg	24 mg/kg
Control **2**	3 ± 1.29 9.6 ± 0.81	2.75 ± 1.60 15.4 ± 2.39	12.6 ± 3.57 35.4 ± 1.60
**11**	24.4 ± 2.56	28.75 ± 6.01	34 ± 4.16

Nifedipine (0.125 mg/kg) caused 50 mmHg drop in arterial blood pressure. ^a^ refers to the number of experiments. Results were significant at *p* < 0.05 according to Mann–Whitney test.

**Table 5:** RMSD values of hit compounds.

Compound No.	2	3a	3b	4	11
RMSD(Å)	0.5694	0.8392	0.7738	0.5678	0.6054

**Table 6:** *In silico* physicochemical properties, drug-likeness and ADME data of the most active compounds.

Cpd. No.	LogP ^a^	M.Wt ^b^	HBA ^c^	HBD ^d^	Lipinski’s Violation	TPSA ^e^	%ABS ^f^	Volumes (A)^3^	S ^g^ (mg/L)	Drug-Likeness Model Score	CaCo2 ^h^	MDCK ^i^	HIA ^j^	BBB ^k^	PPB ^l^	CYP3A4 Inhibition	CYP2D6 Inhibition
**2**	1.82	272.31	6	3	0	90.14	77.90	248.72	77.22	0.16	20.44	77.38	92.60	0.67	69.58	Non	Non
**11**	3.17	336.39	6	0	0	69.92	84.87	310.77	3.06	0.06	33.73	18.32	98.72	1.58	88.16	inhibitor	Non

^a^Log P: logarithm of compound partition coefficient between n-octanol and water. ^b^ M.Wt: molecular weight. ^c^ HBA: number of hydrogen bond acceptors. ^d^ HBD: number of hydrogen bond donors. ^e^ TPSA: polar surface area. ^f^ %ABS: percentage of absorption. ^g^ S: aqueous solubility. ^h^ CaCo2: permeability through cells derived from human colon adenocarcinoma. ^I^ MDCK: permeability through Madin–Darby canine kidney cells. ^j^ HIA: human intestinal absorption. ^k^ BBB: blood–brain barrier penetration. ^l^ PPB: plasma protein binding.

## Data Availability

Not applicable.

## Supplementary Materials

The following supporting information can be downloaded at: https://www.mdpi.com/article/10.3390/molecules27072240/s1; Figures S1–S28: Spectra of compounds 2-14; Figures S29–S31: Pharmacophore elucidation.
