# Supplementary material for: Design, Synthesis, Pharmacodynamic and In Silico Pharmacokinetic Evaluation of Some Novel Biginelli-Derived Pyrimidines and Fused Pyrimidines as Calcium Channel Blockers
Source: Molecules. 2022 Mar 30;27(7):2240. doi: 10.3390/molecules27072240 (PMC9000669; doi:10.3390/molecules27072240)
Supplement: Supplementary file 1 [file molecules-27-02240-s001.zip › molecules-1612438-supplementary.pdf]

molecules

Supporting Information

Design, Synthesis, Pharmacodynamic and *In Silico* Pharmacokinetic Evaluation of Some Novel Biginelli-Derived Pyrimidines and Fused Pyrimidines as Calcium Channel Blockers

Ahmed M. Farghaly [1,*], Ola H. Rizk [1,2], Inas Darwish [3,4], Manal Hamza [3], Mezna Saleh Altowyan [5], Assem Barakat [6,7] and Mohamed Teleb [1,*]

[1] Department of Pharmaceutical Chemistry, Faculty of Pharmacy, Alexandria University, Alexandria 21521, Egypt
[2] Department of Pharmaceutical Chemistry, Faculty of Pharmacy, Pharos University, Alexandria 21521, Egypt; olarizk@yahoo.com
[3] Department of Clinical Pharmacology, Faculty of Medicine, Alexandria University, Alexandria 21521, Egypt; inas.darwish@alexmed.edu.eg or inasdarwish@gmail.com (I.D.); manalhamza@gmail.com (M.H.)
[4] Department of Pharmacology and Therapeutics, Faculty of Pharmacy, Pharos University, Alexandria 21521, Egypt
[5] Department of Chemistry, College of Science, Princess Nourah bint Abdulrahman University, P.O. Box 84428, Riyadh 11671, Saudi Arabia; msaltowyan@pnu.edu.sa
[6] Department of Chemistry, College of Science, King Saud University, P.O. Box 2455, Riyadh 11451, Saudi Arabia; ambarakat@ksu.edu.sa
[7] Department of Chemistry, Faculty of Science, Alexandria University, P.O. Box 426, Ibrahimia, Alexandria 21321, Egypt.
* Correspondence: farghalyahmedau@gmail.com (A.M.F.); mohamed.t.ismail@alexu.edu.eg (M.T.)

Table of content

Content	Page
Spectra of compounds 2-14	S1-S30
Pharmacophore elucidation	S30-S32

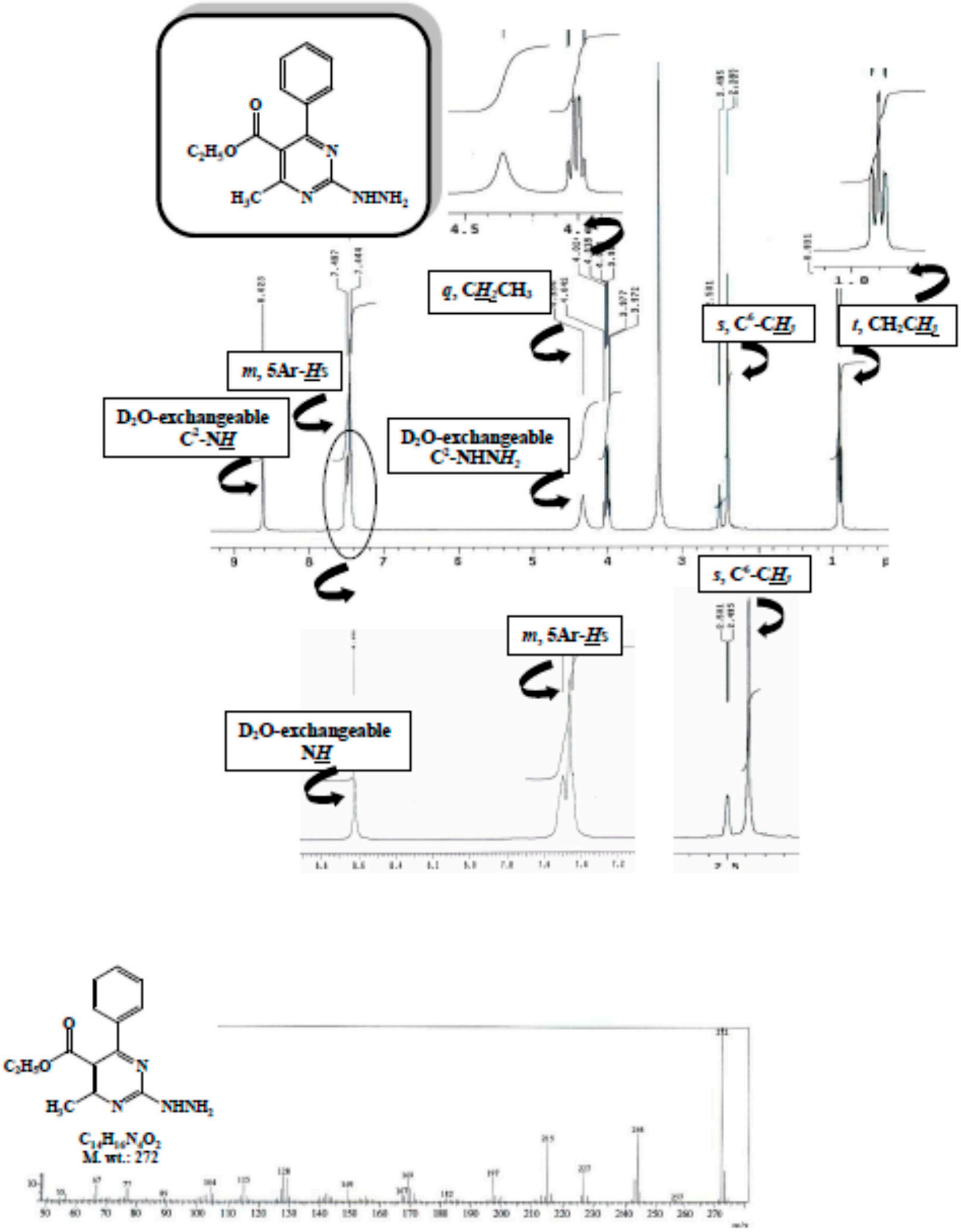

Figure S1. ^{1}H NMR and mass spectra of compound **2**

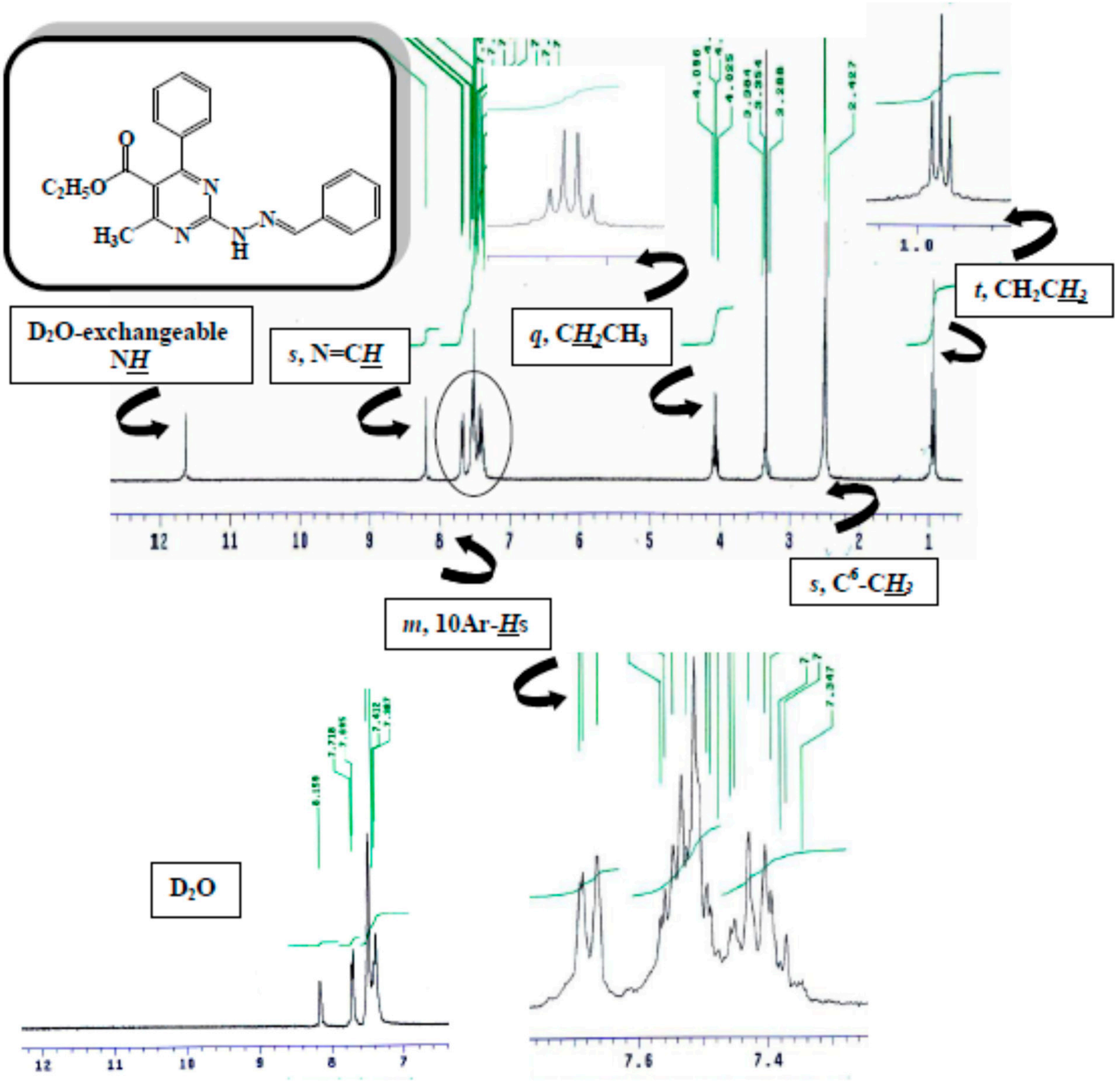

Figure S2. ^{1}H NMR spectrum of compound **3a**

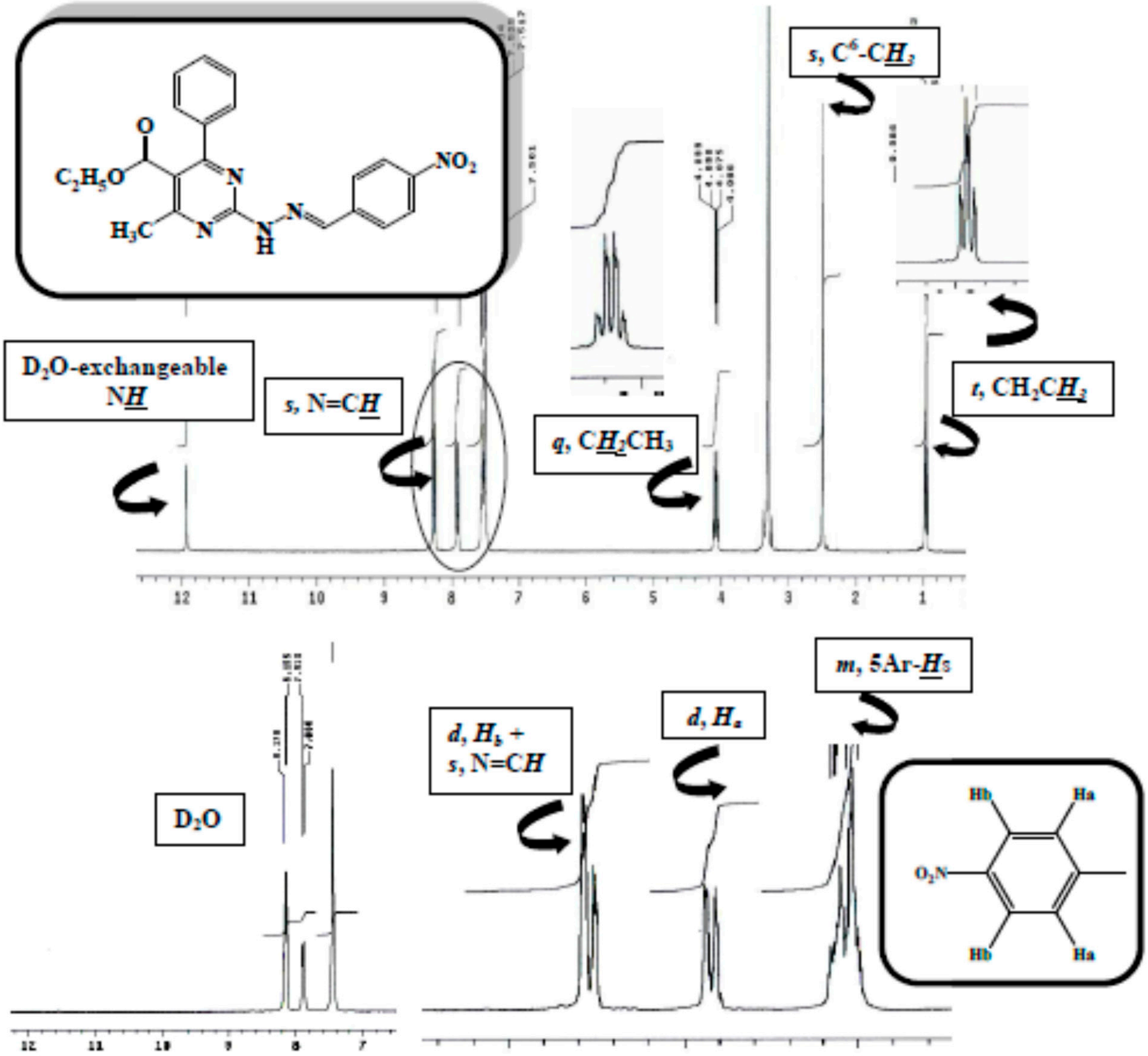

Figure S3. ^{1}H NMR spectrum of compound **3b**

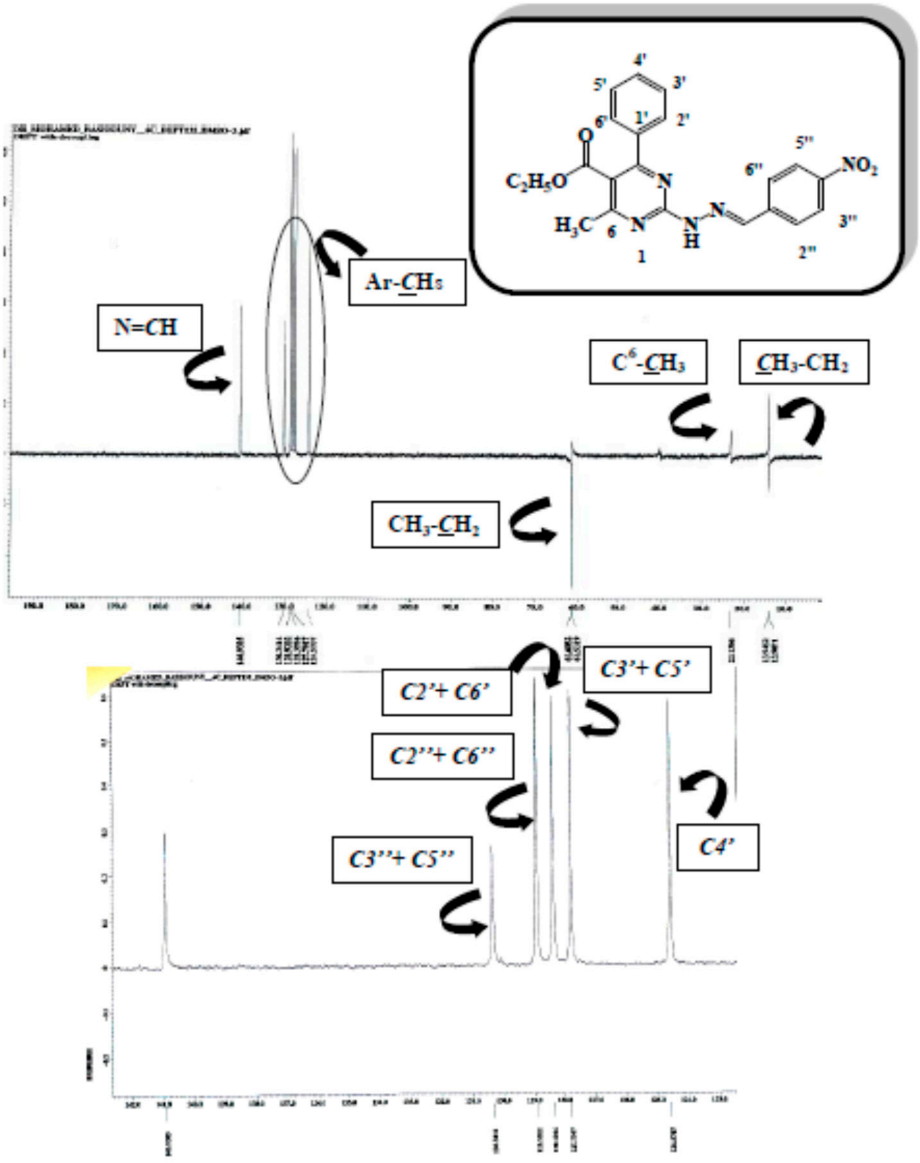

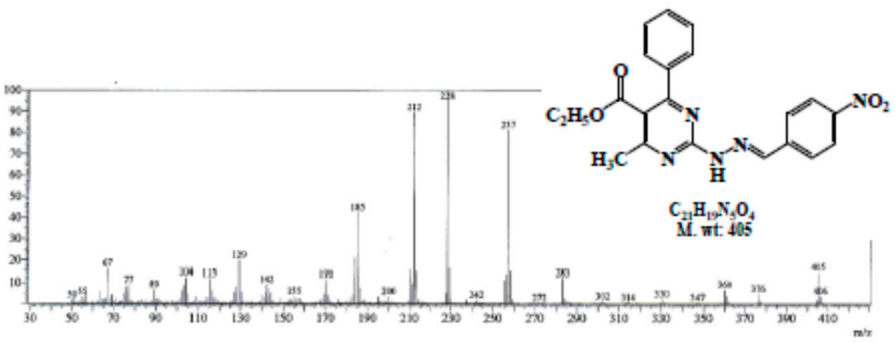

Figure S4. ^{13}C NMR and mass spectra of compound **3b**

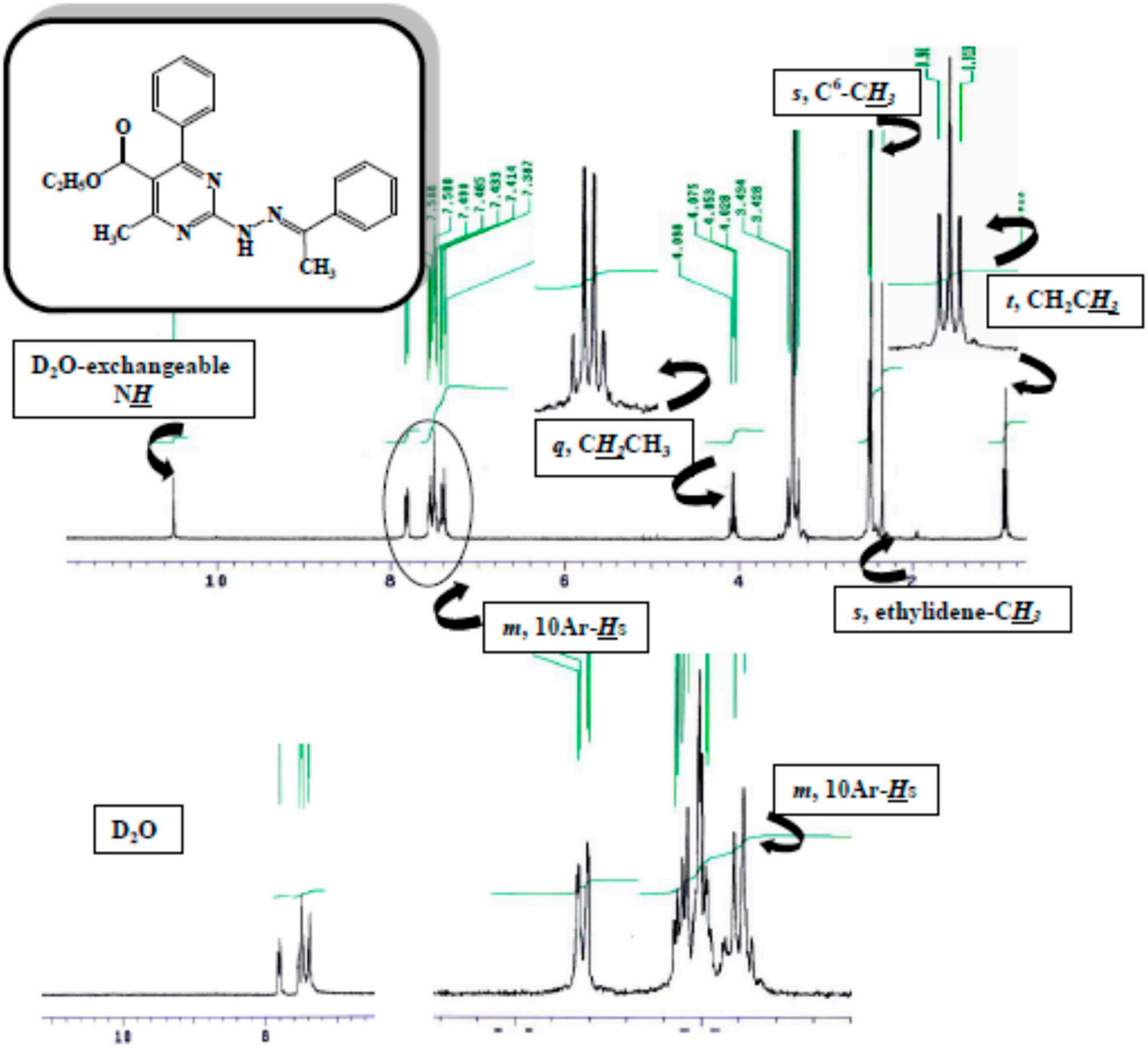

Figure S5. ^{1}H NMR spectrum of compound **4**

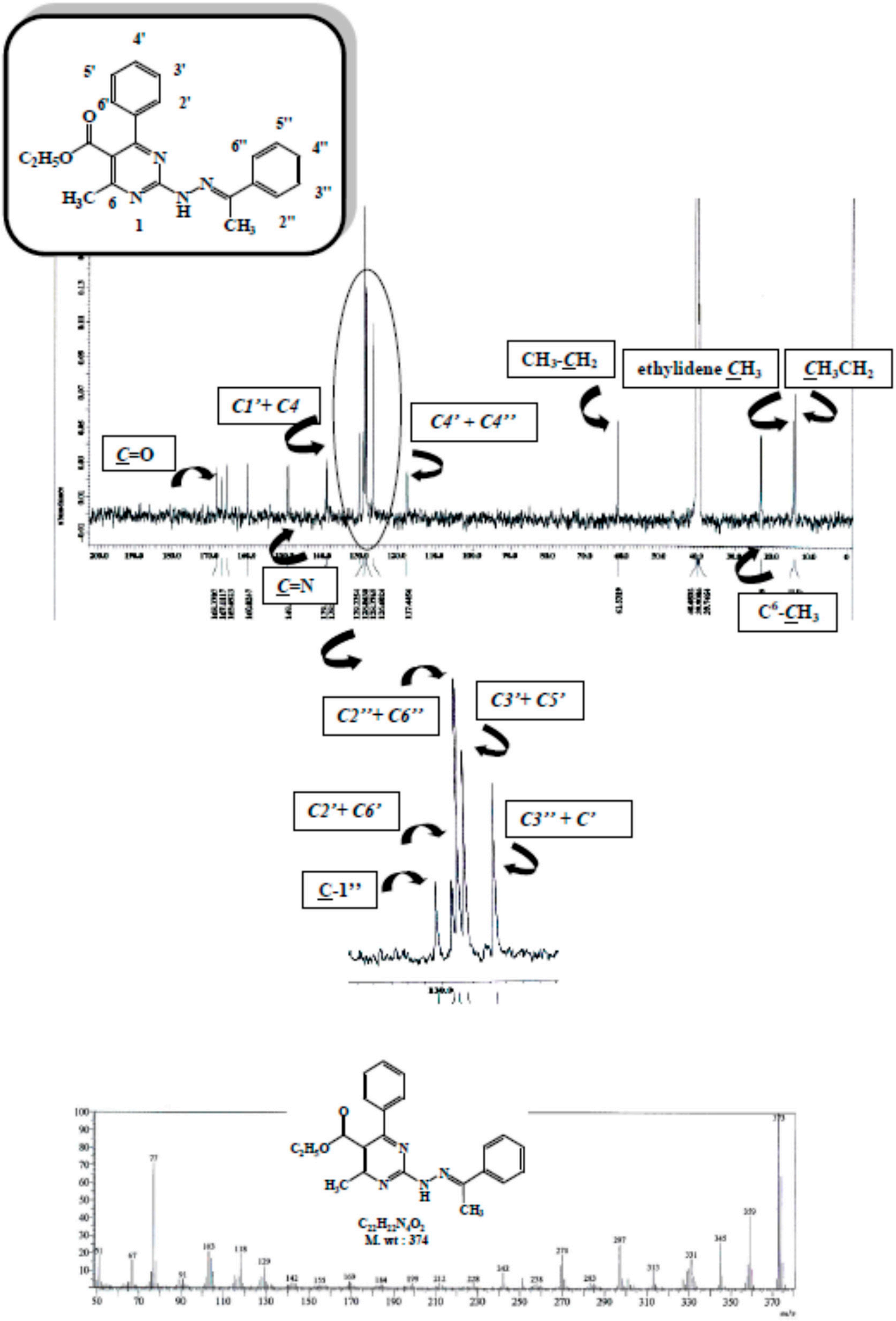

Figure S6. ^{13}C NMR and mass spectra of compound **4**

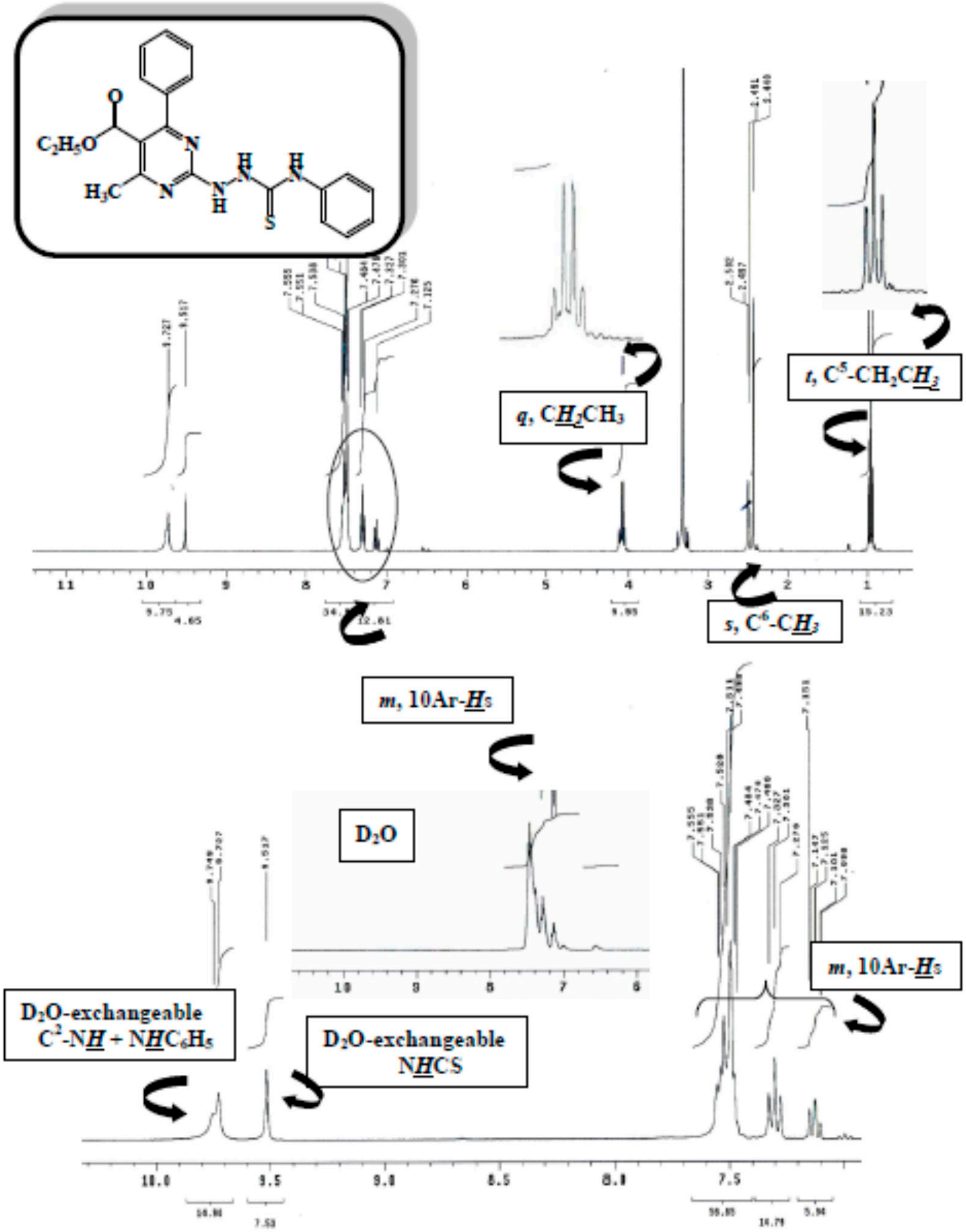

Figure S7. ^{1}H NMR spectrum of compound **5a**

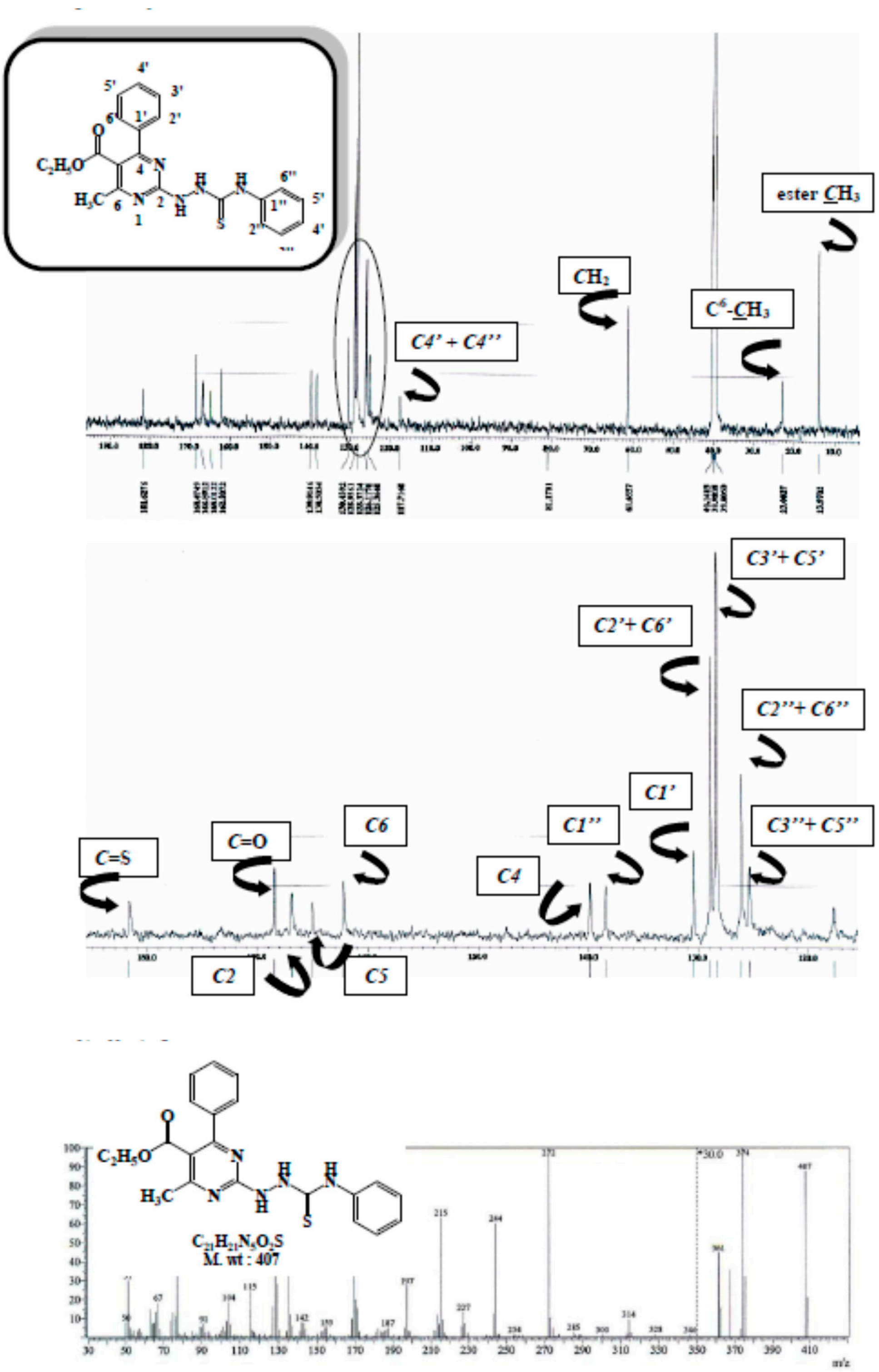

Figure S8. ^{13}C NMR and mass spectra of compound **5a**

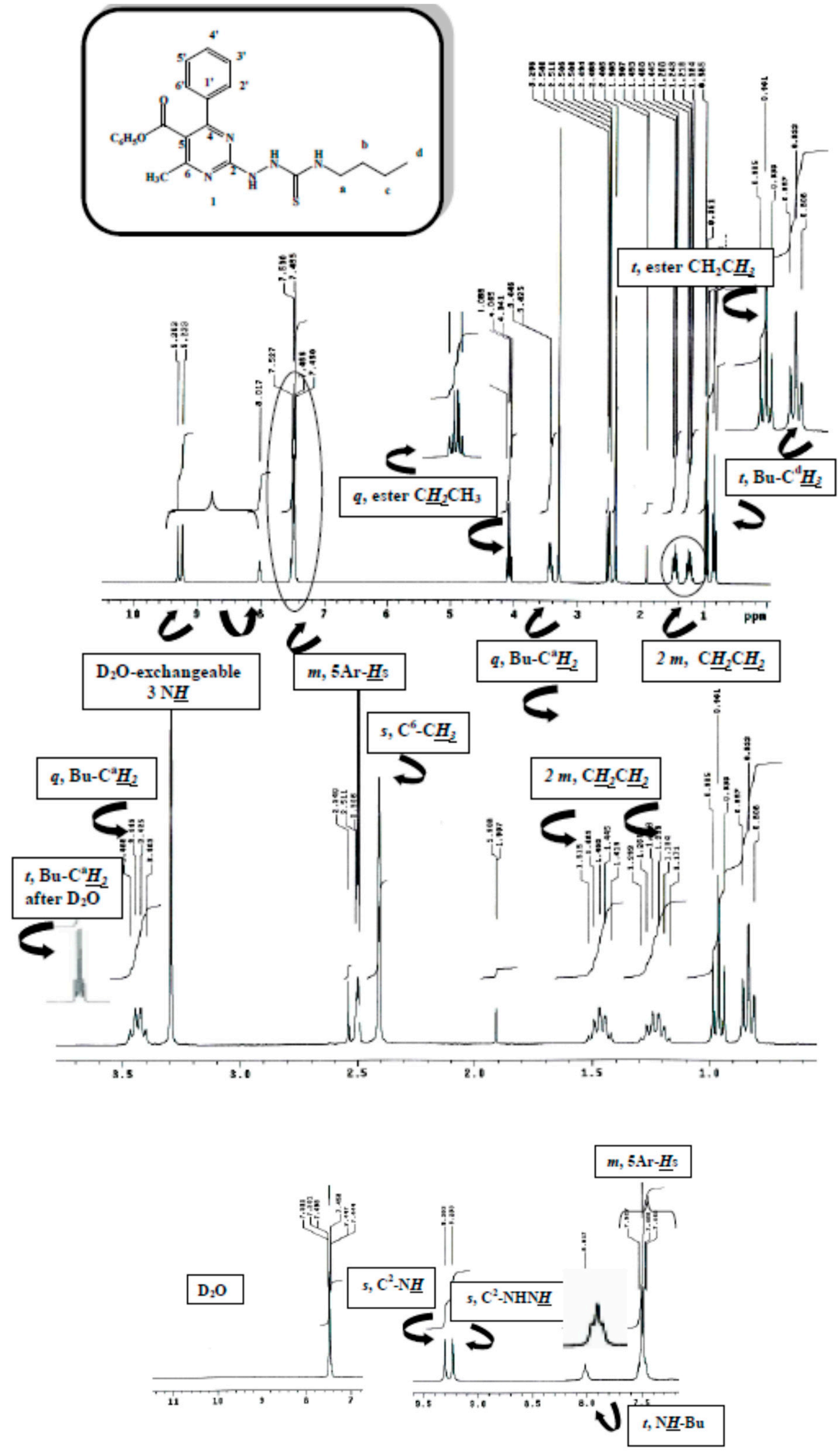

Figure S9. ^{1}H NMR spectrum of compound **5b**

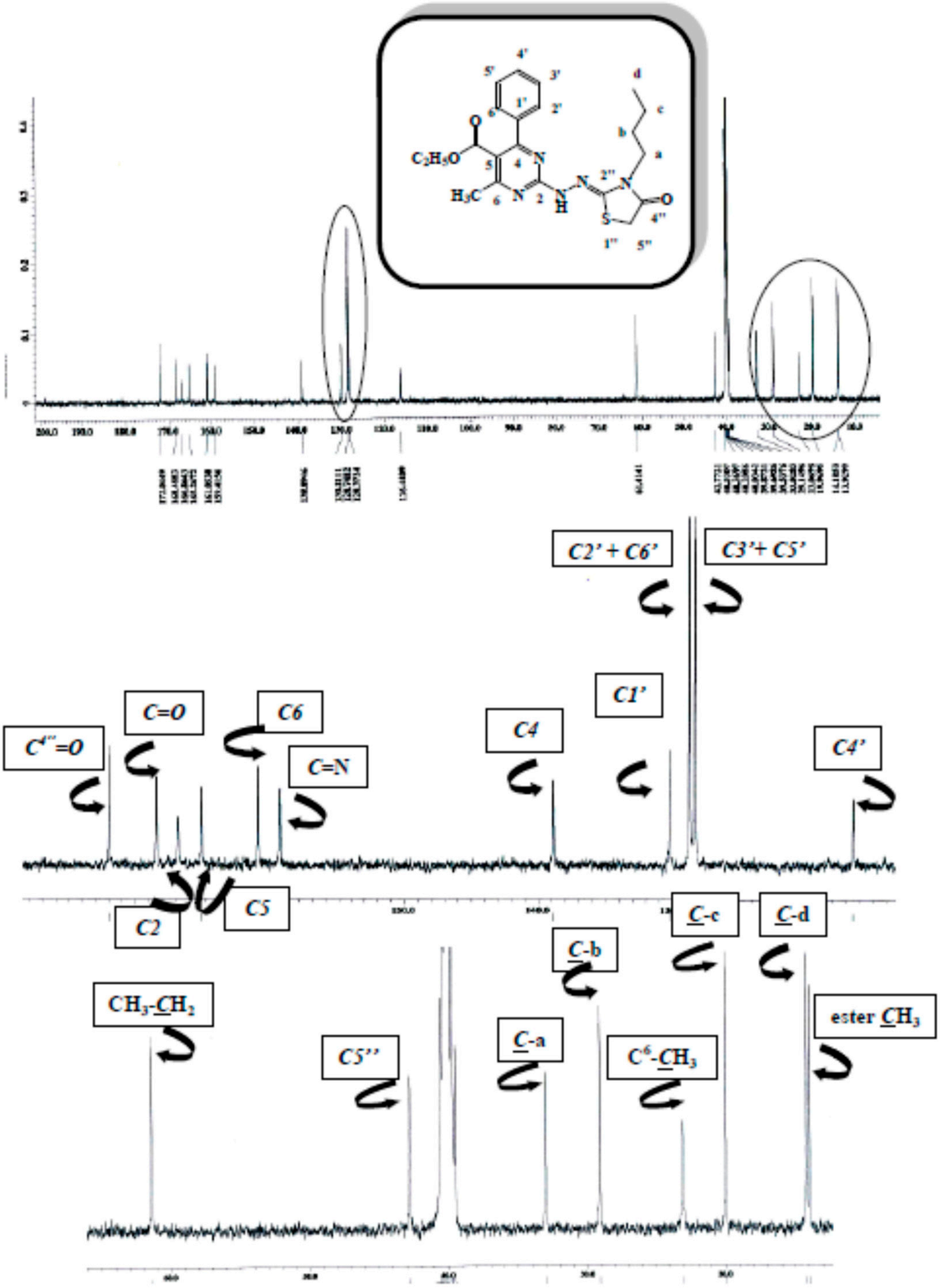

Figure S10. ^{13}C NMR spectrum of compound **6**

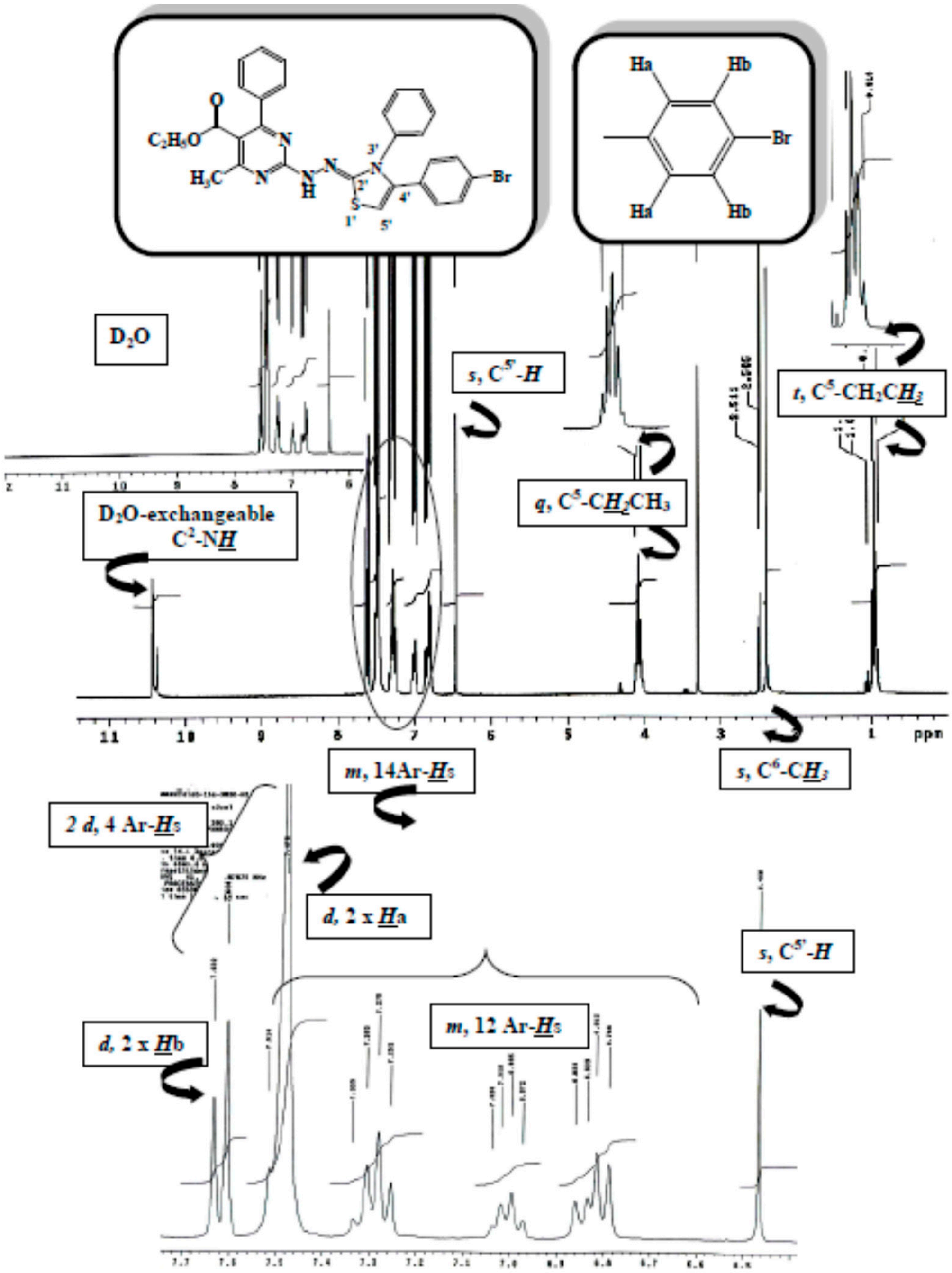

Figure S11. ^{1}H NMR spectrum of compound **7**

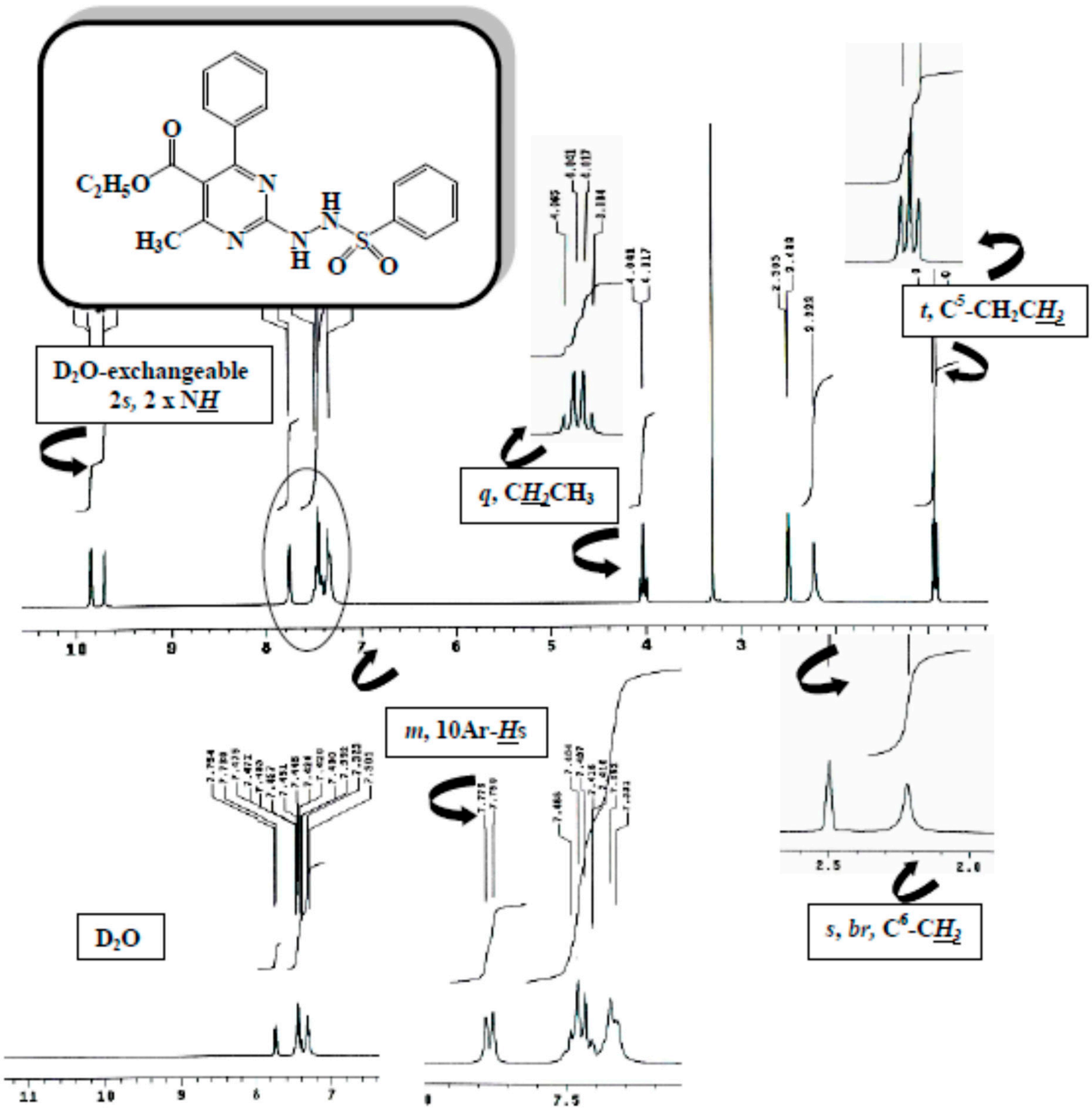

Figure S12. ^{1}H NMR spectrum of compound **8a**

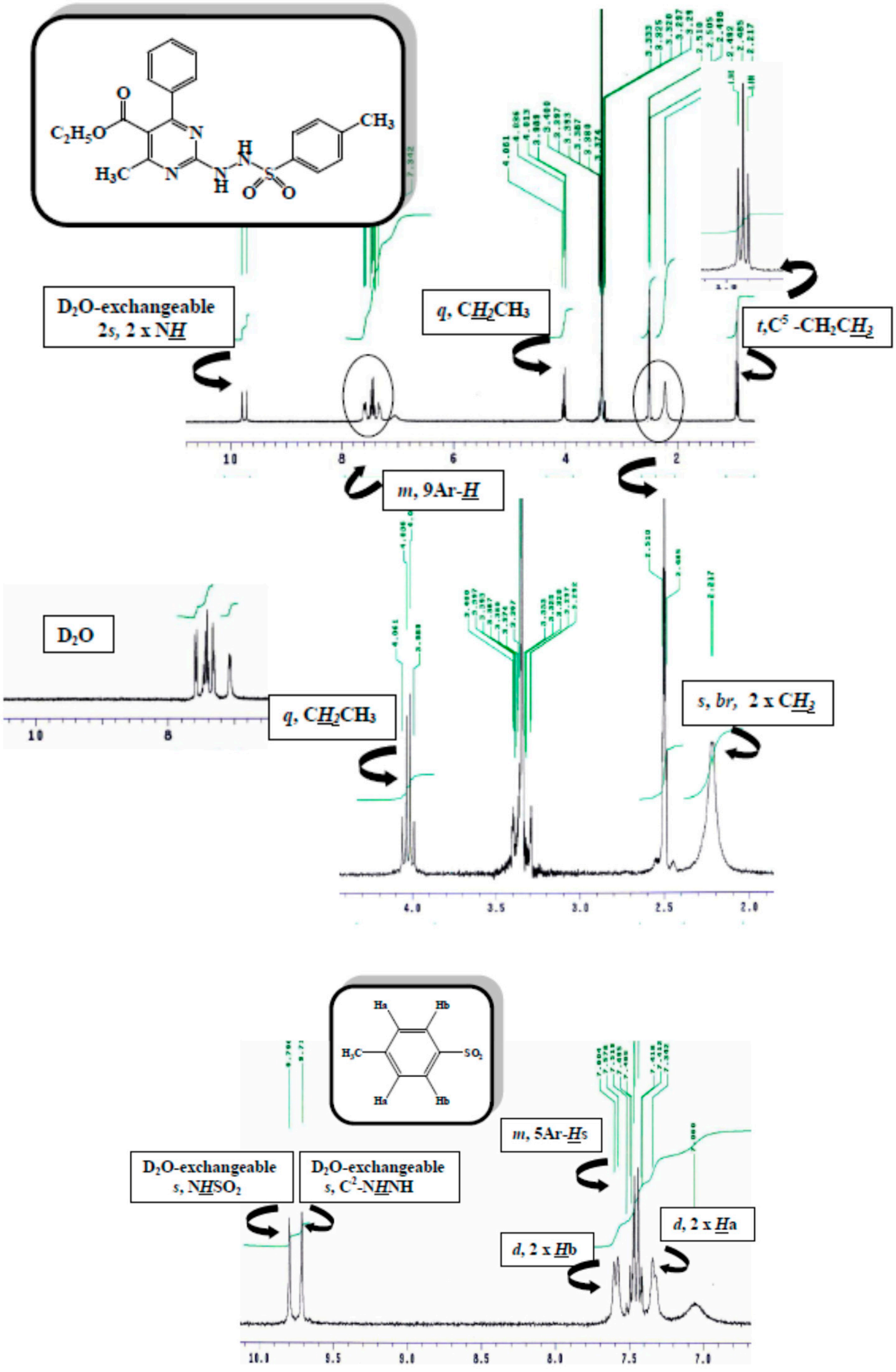

Figure S13. ^{1}H NMR spectrum of compound **8b**

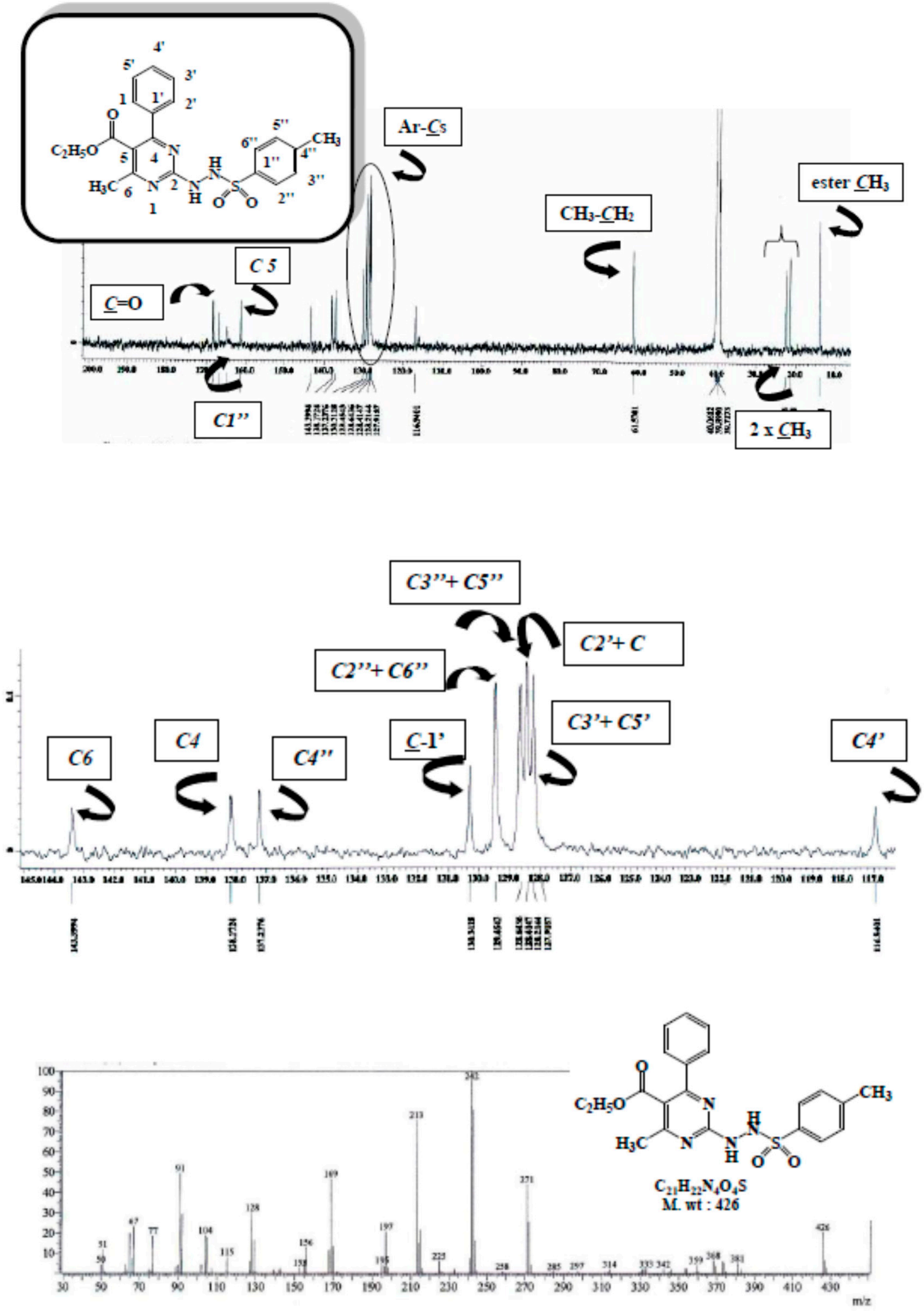

Figure S14. ^{13}C NMR and mass spectra of compound **8b**

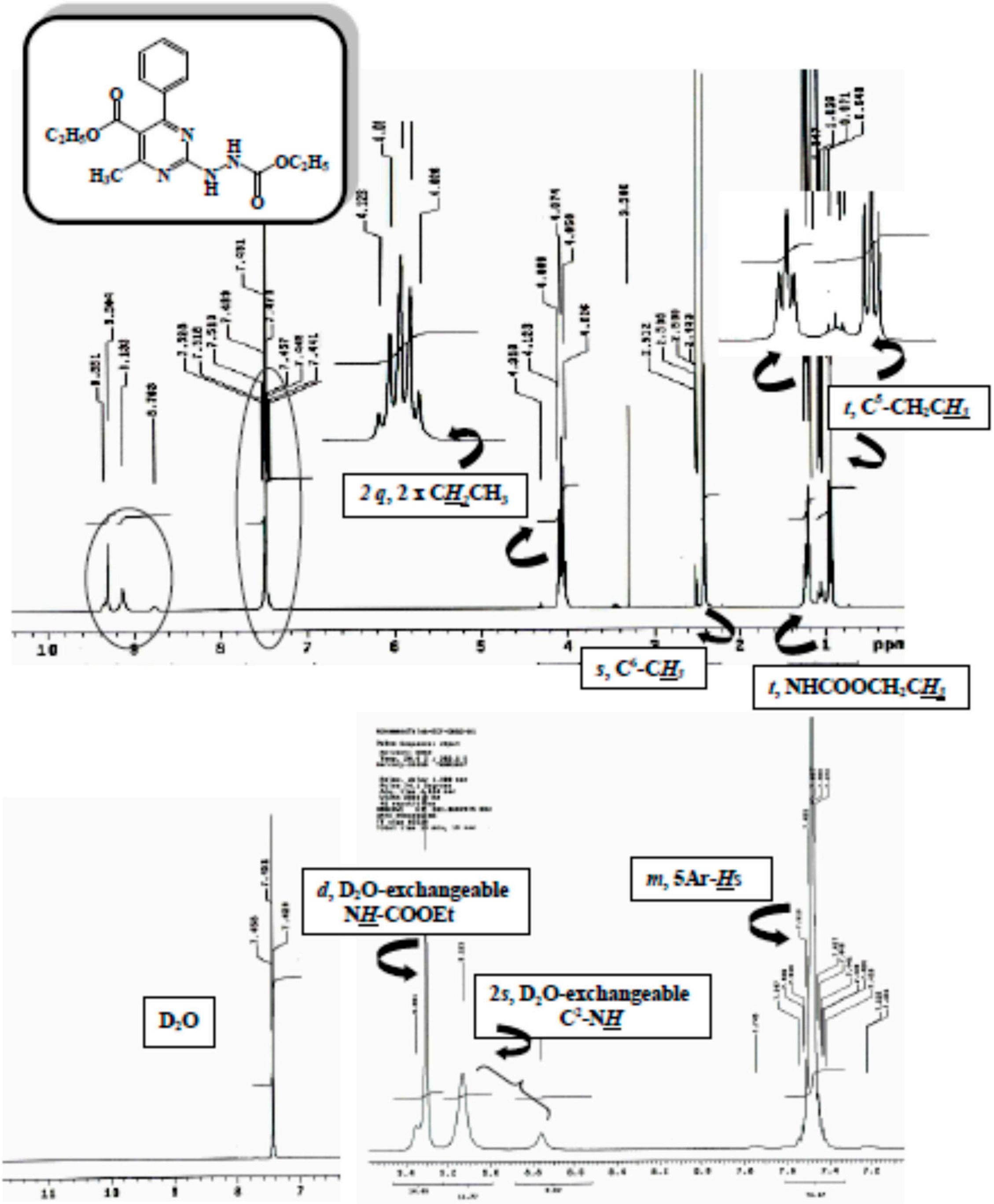

Figure S15. ^{1}H NMR spectrum of compound **9**

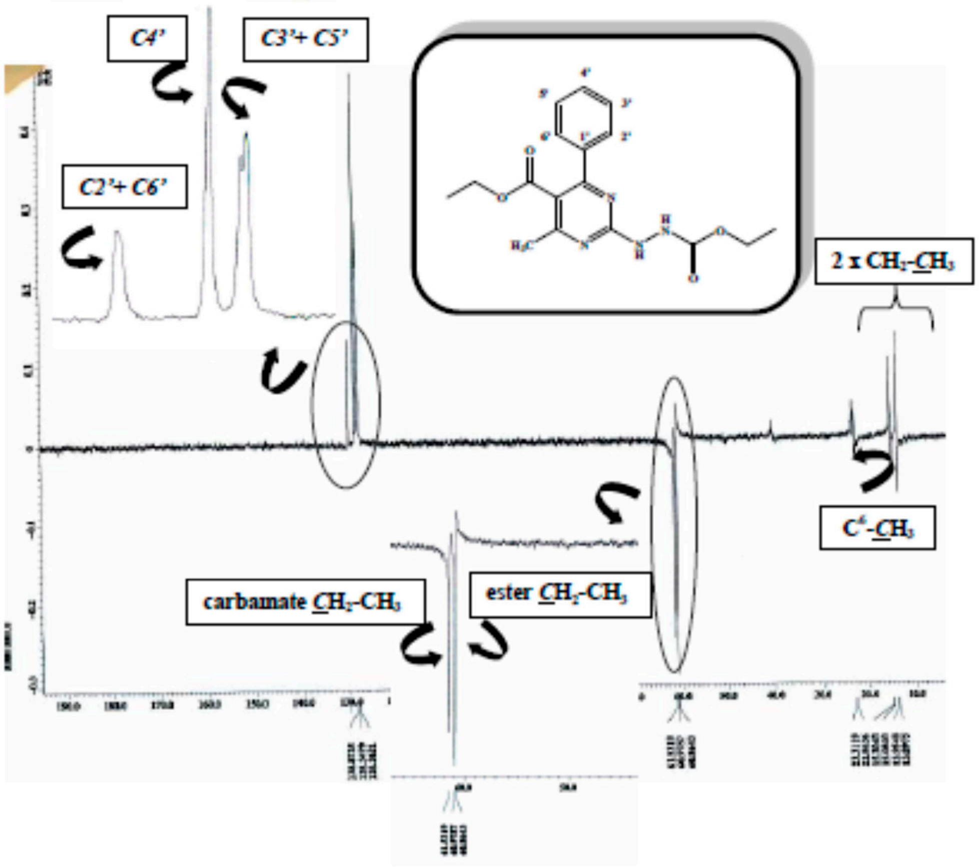

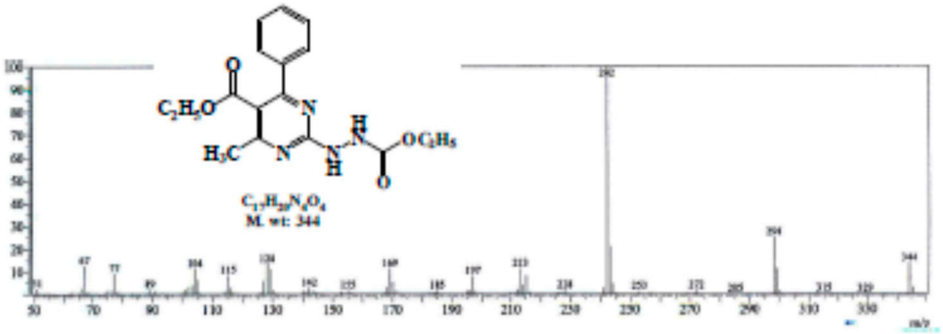

Figure S16. ^{13}C DEPT NMR and mass spectra of compound **9**

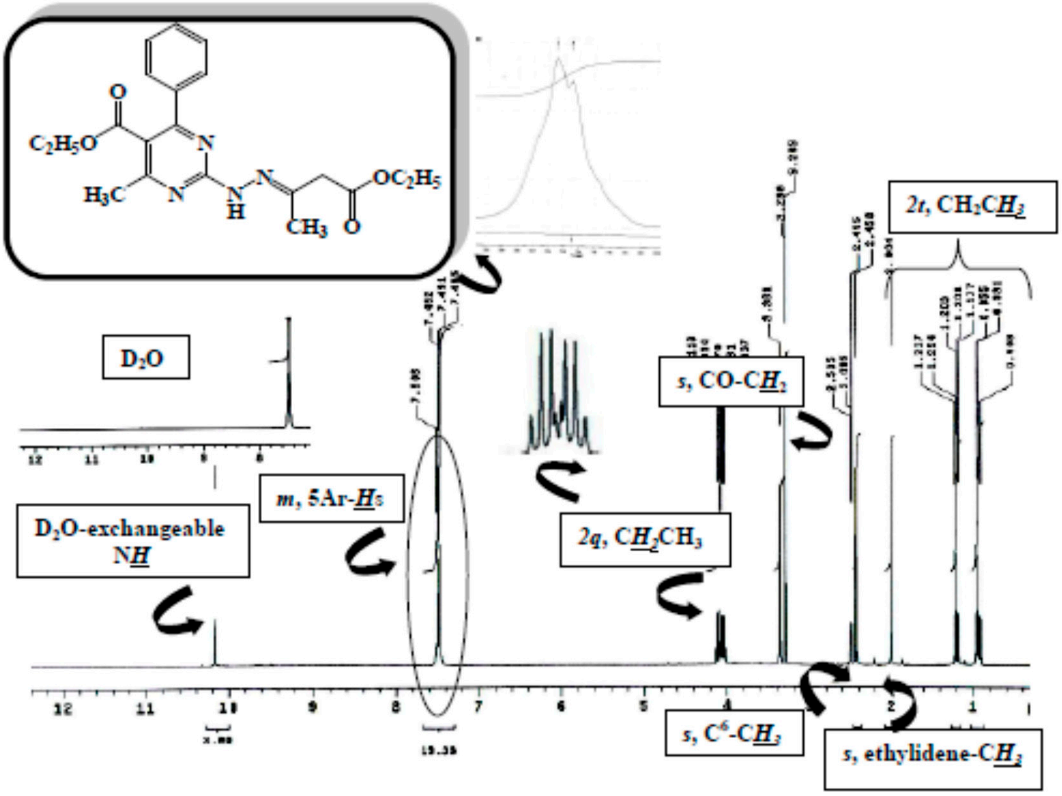

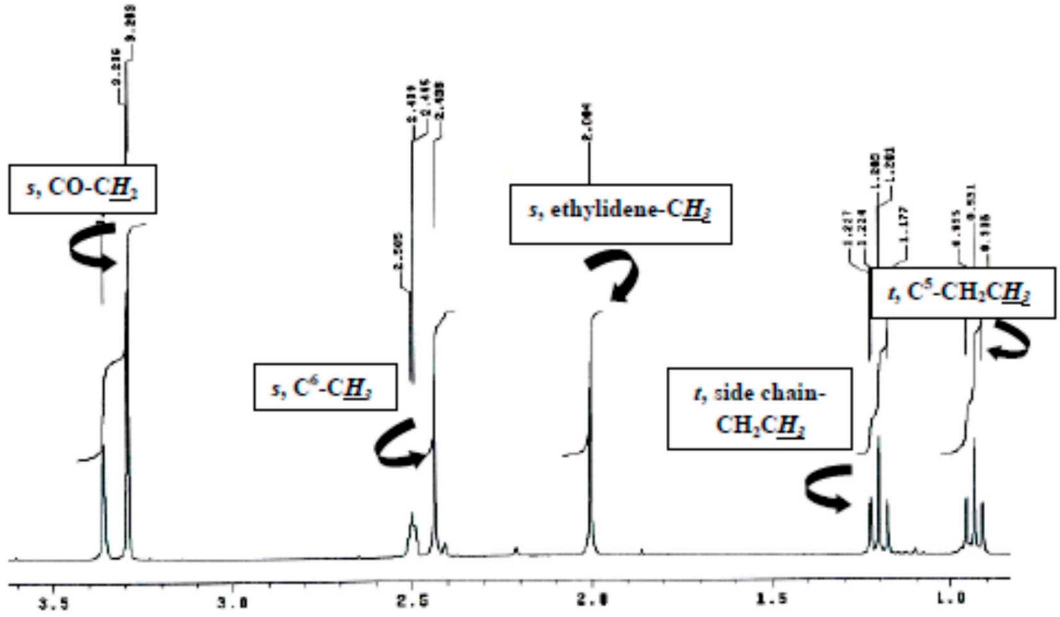

Figure S17. ^{1}H NMR spectrum of compound **10**

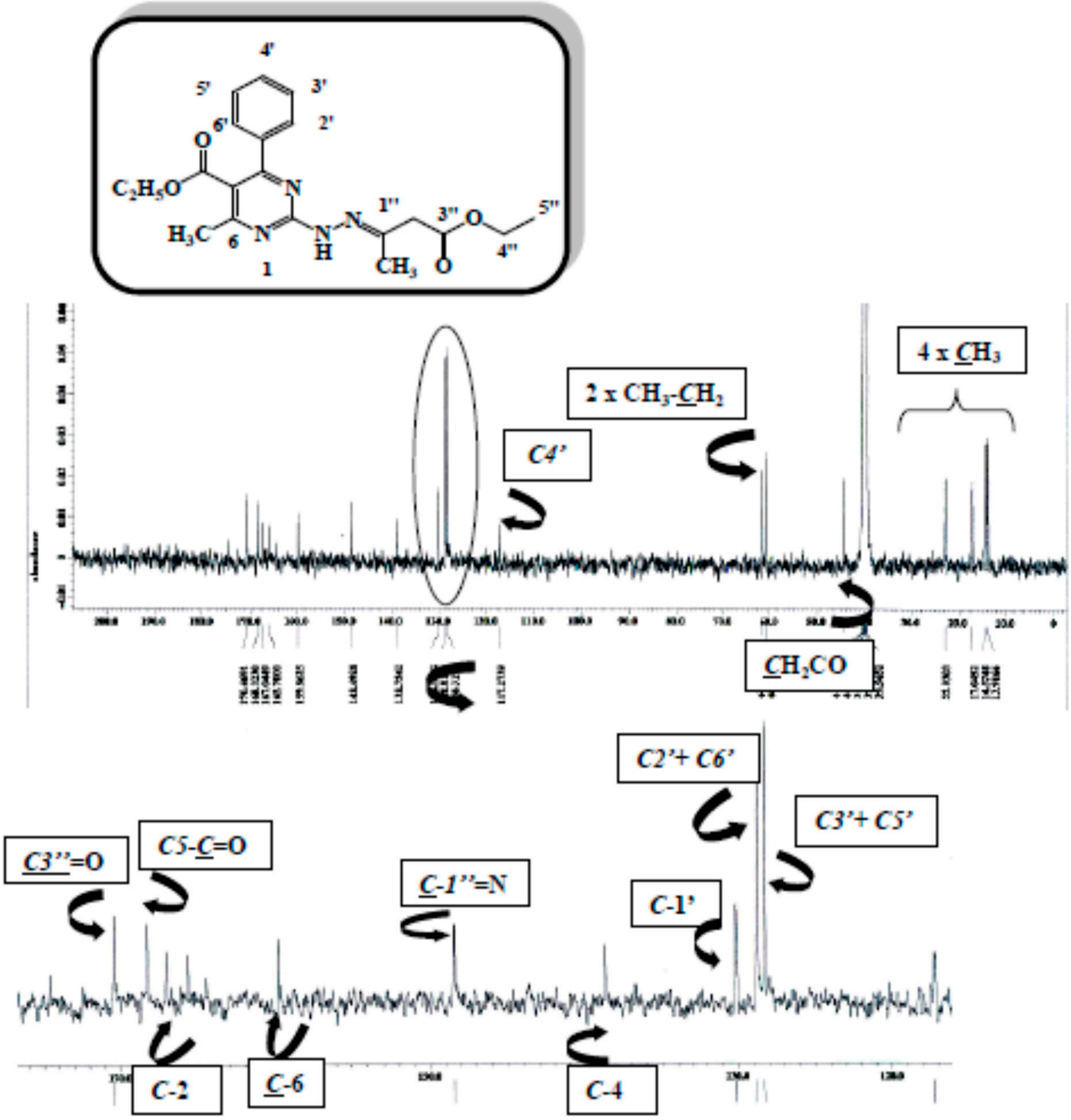

Figure S18. ^{13}CNMR spectrum of compound **10**

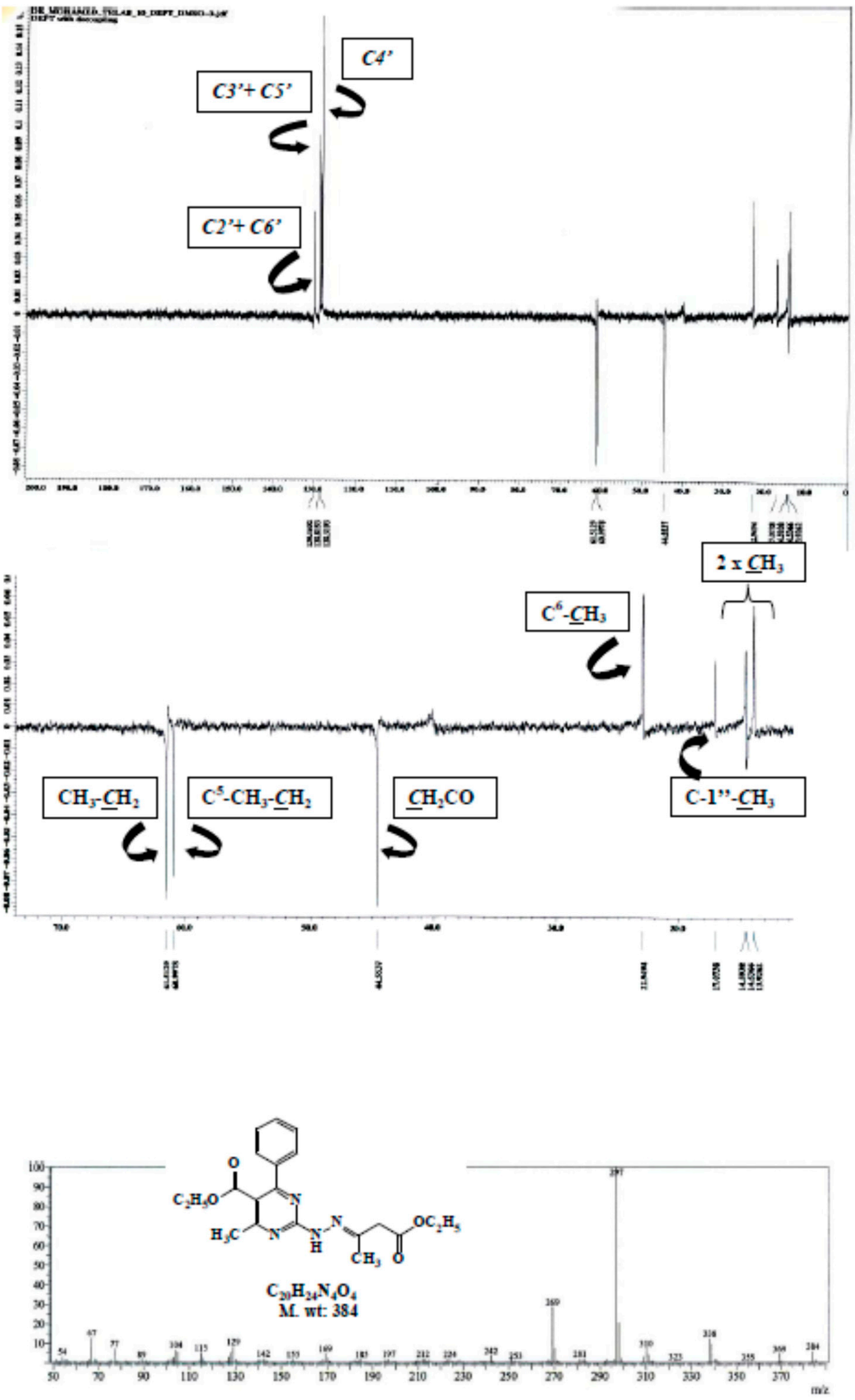

Figure S19. ^{13}C DEPT NMR and mass spectra of compound **10**

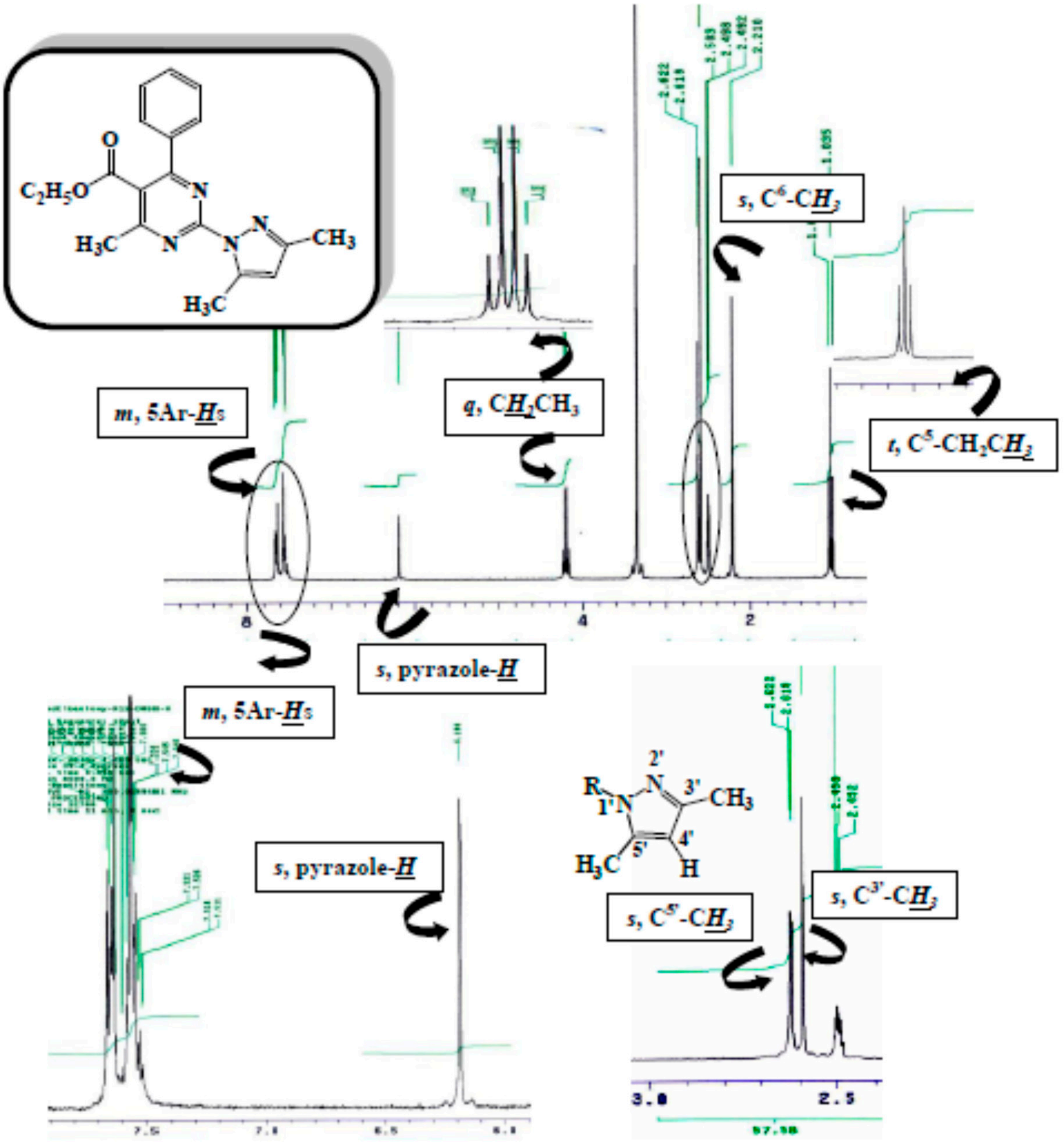

Figure S20. ^{1}H NMR spectrum of compound **11**

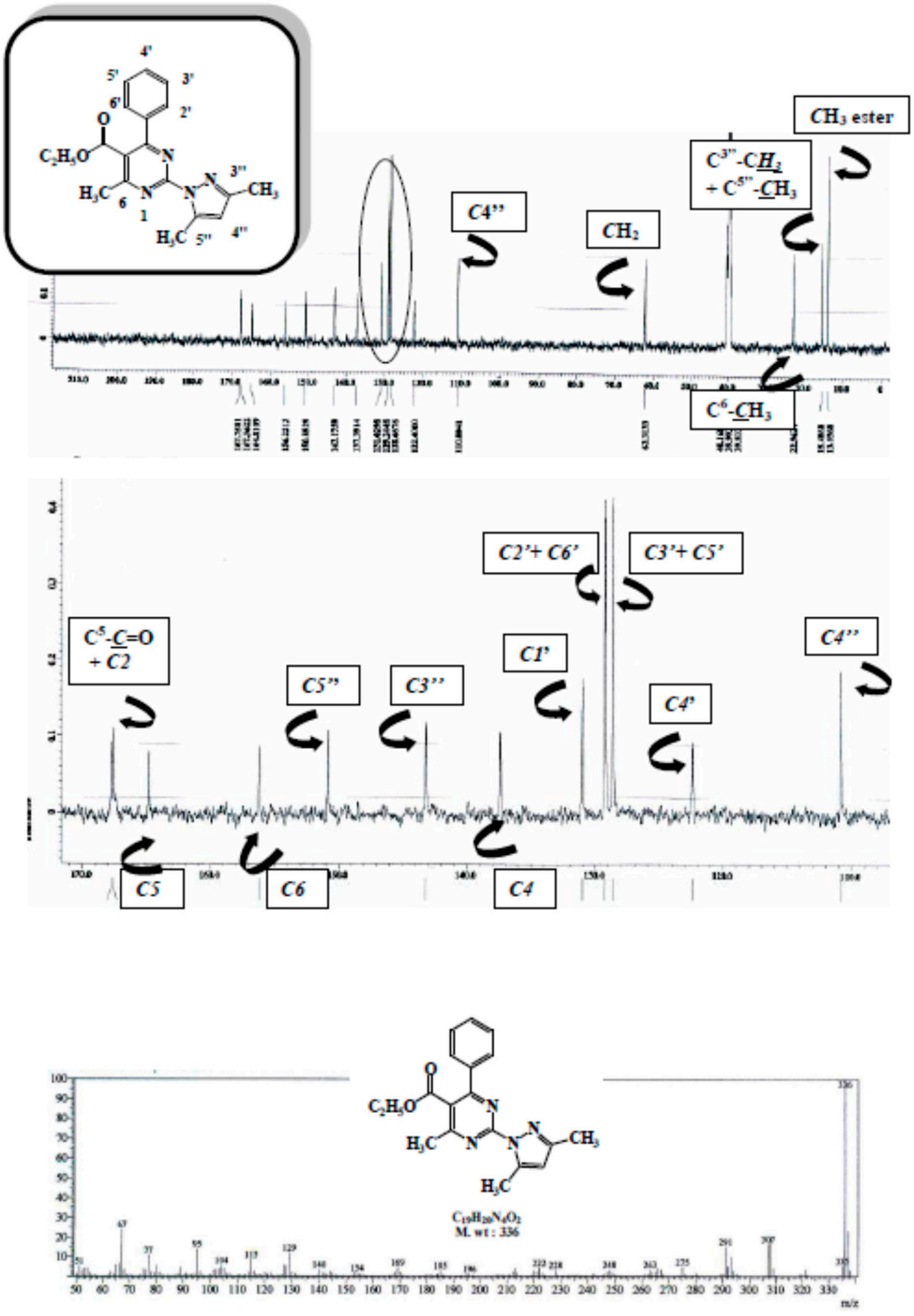

Figure S21. ^{13}C NMR and mass spectra of compound **11**

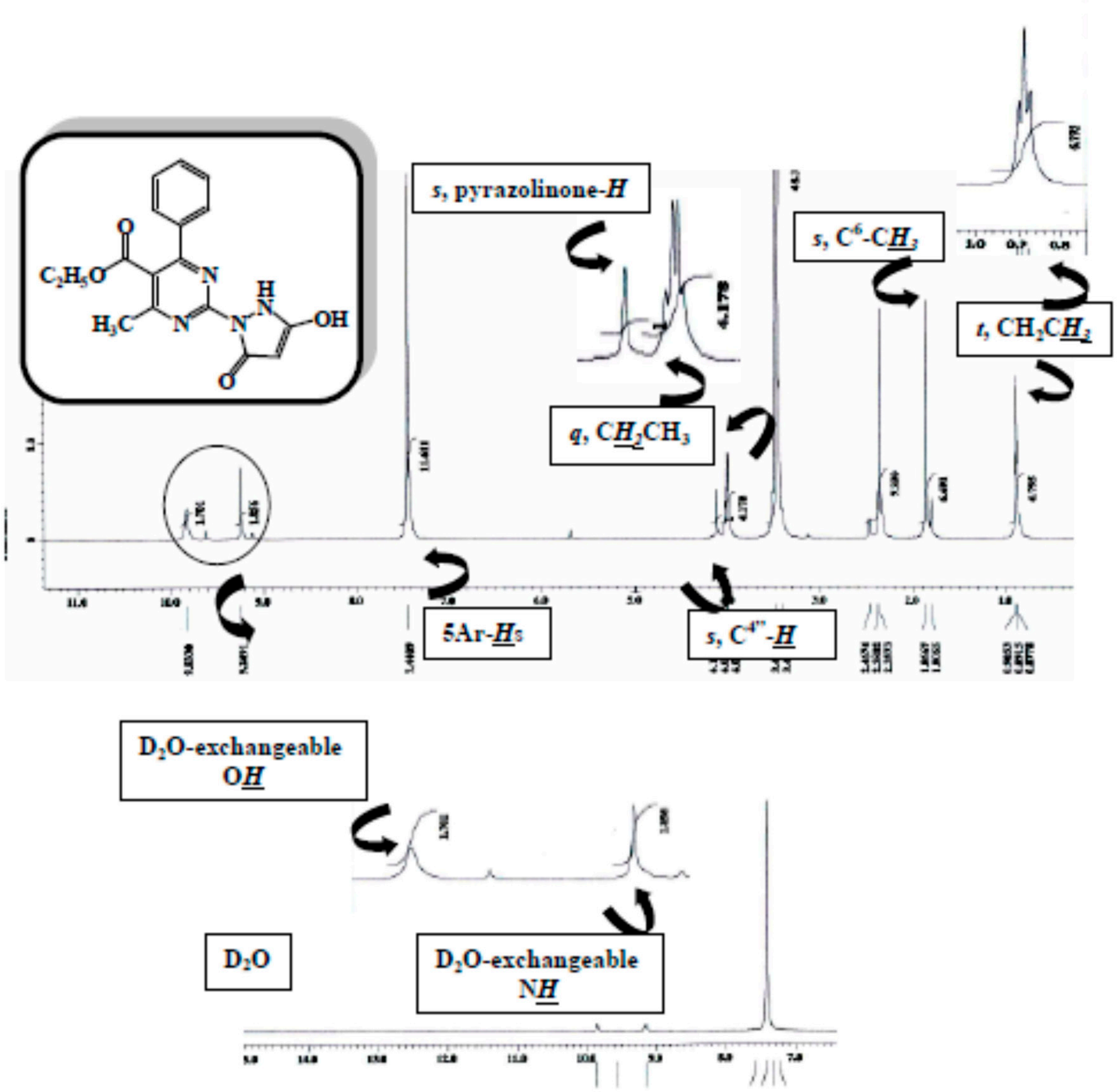

Figure S22. ^{1}H NMR spectrum of compound **12**

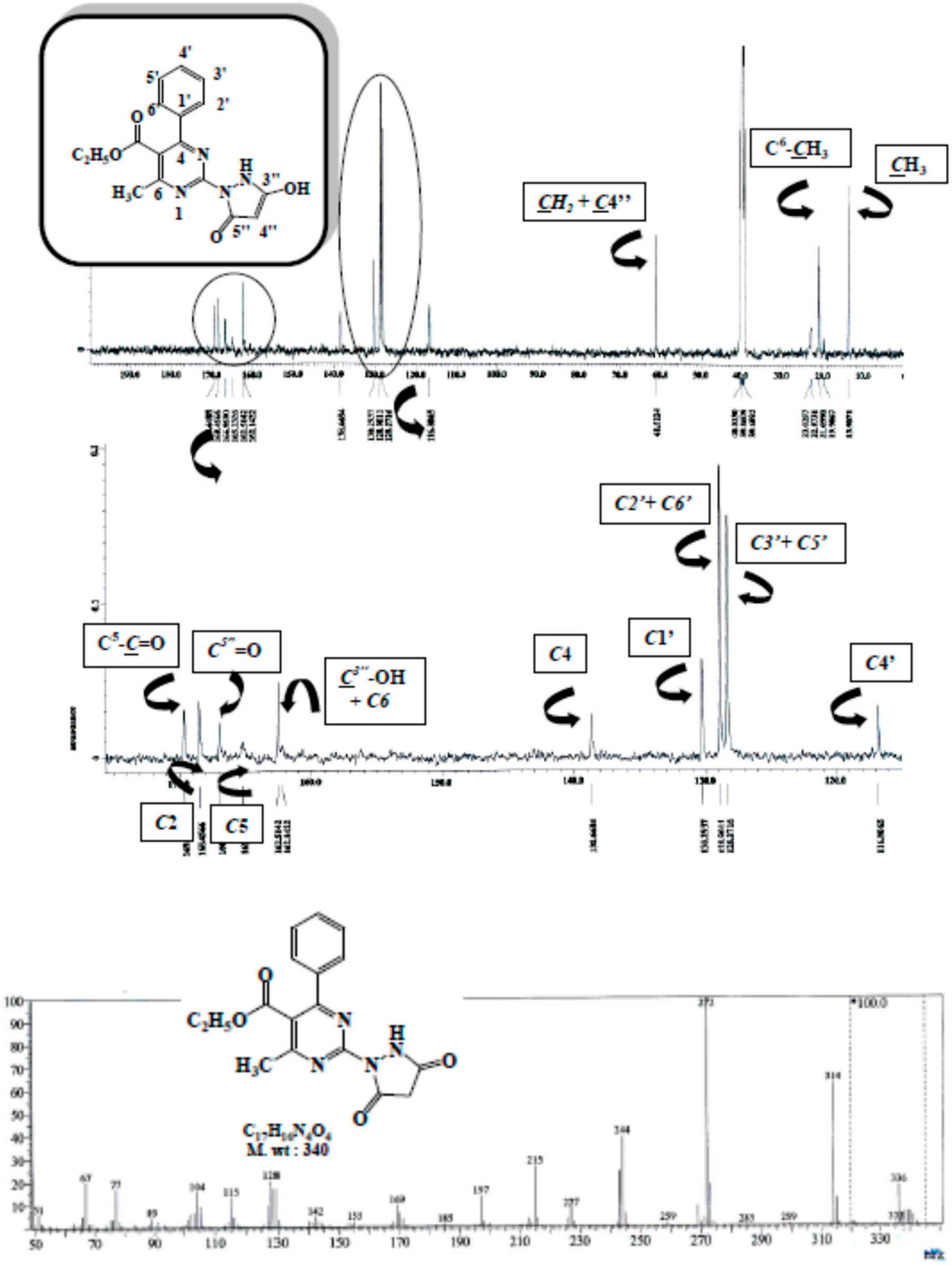

Figure S23. ^{13}C NMR and mass spectra of compound **12**

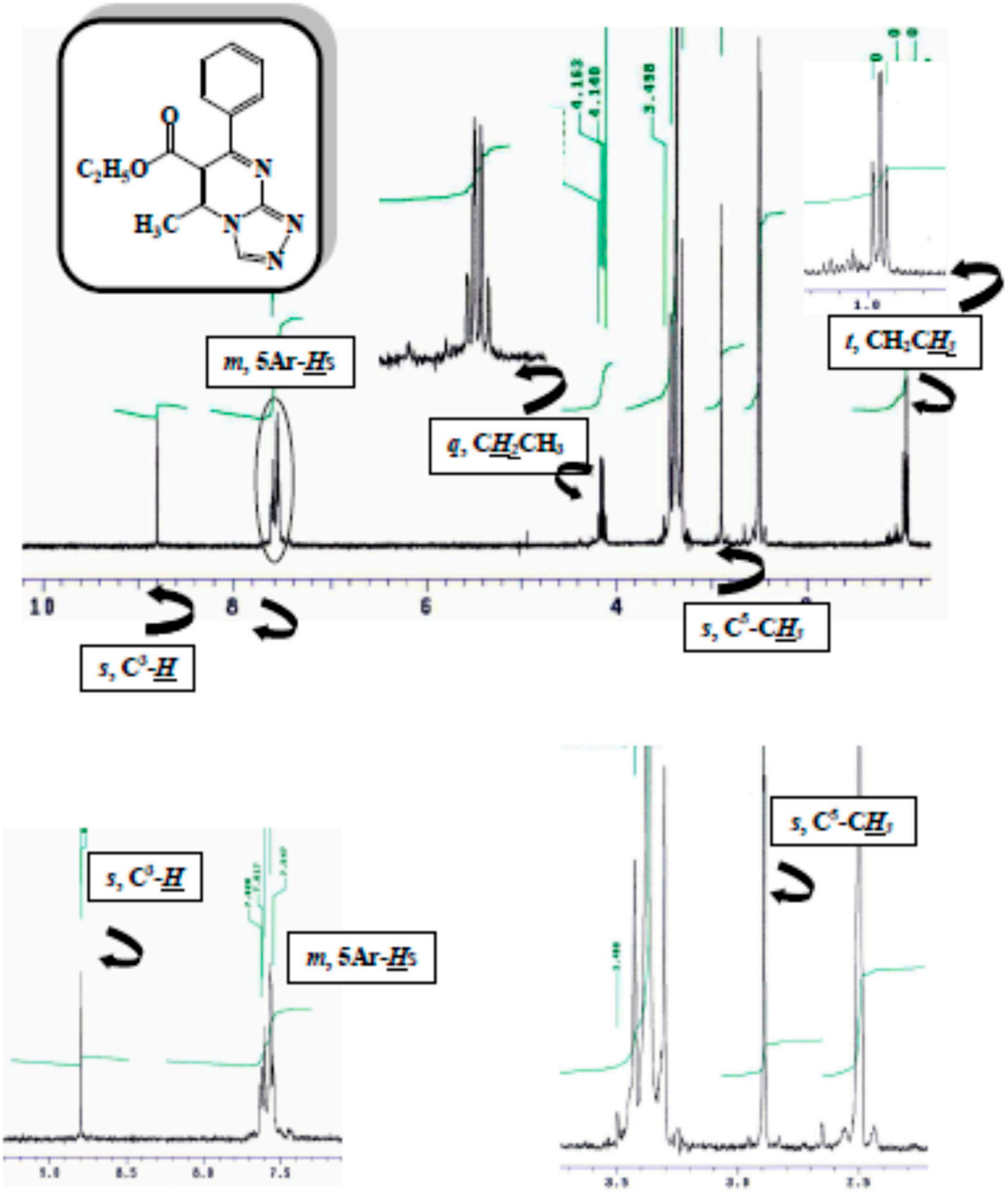

Figure S24. ^{1}H NMR spectrum of compound **13**

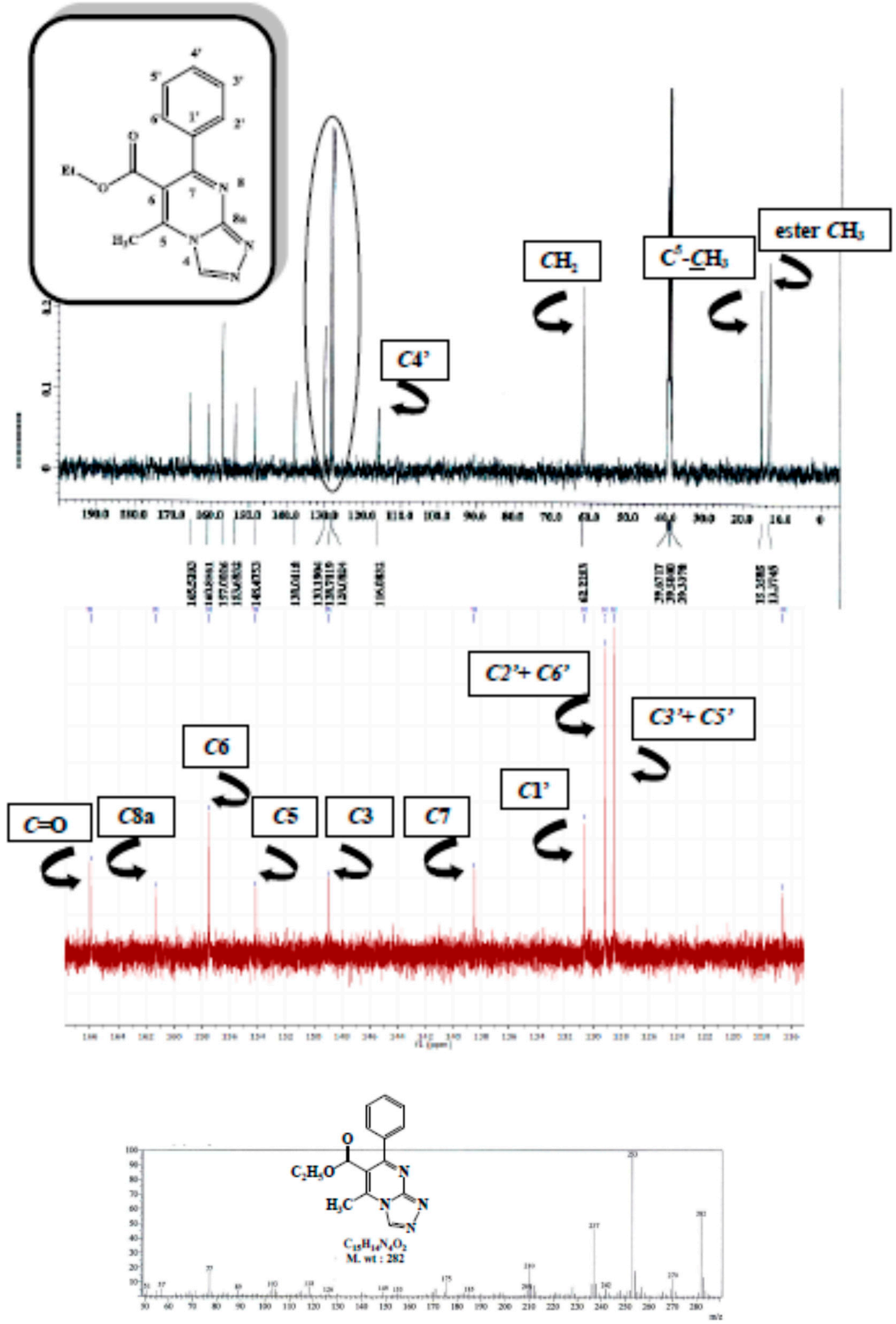

Figure S25. ^{13}C NMR and mass spectra of compound **13**

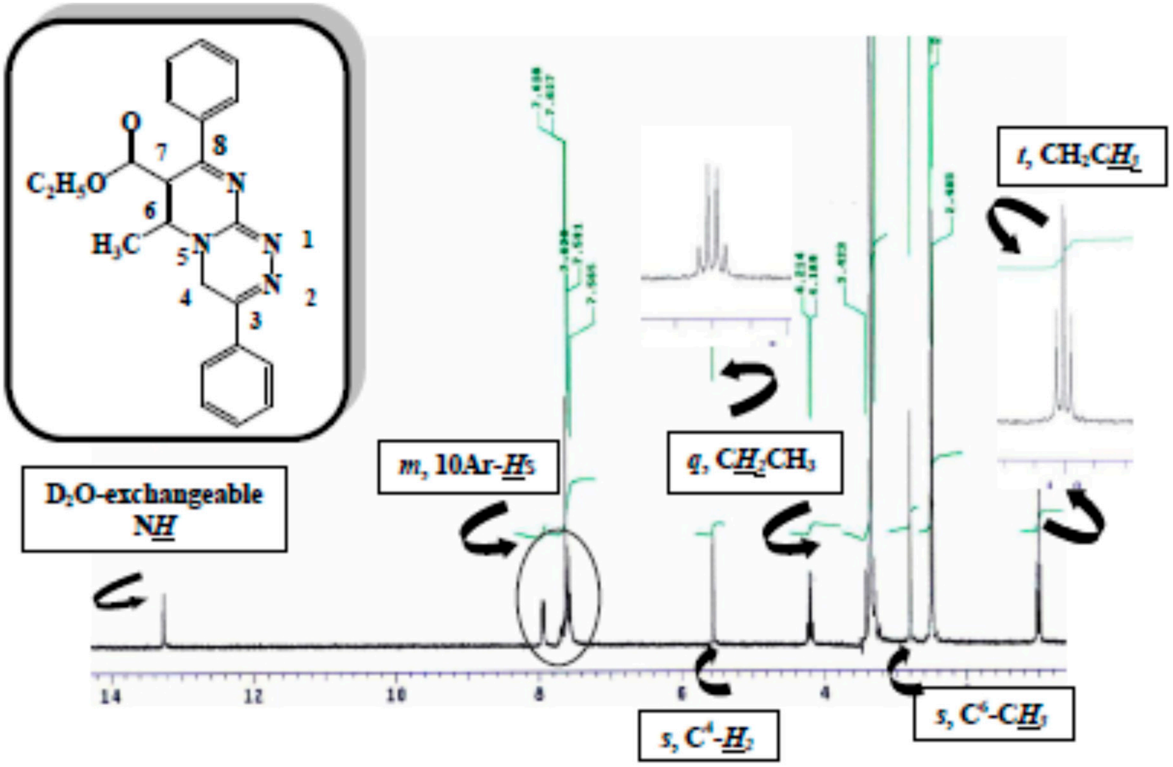

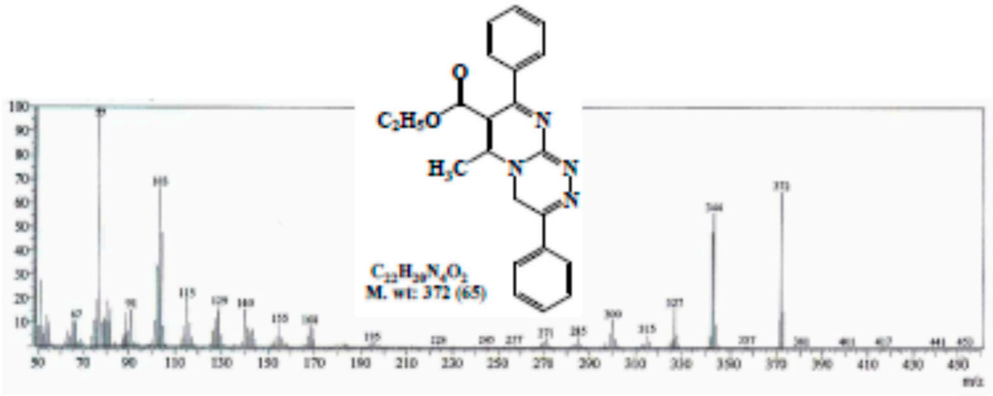

Figure S26. ^{1}H NMR and mass spectra of compound **14a**

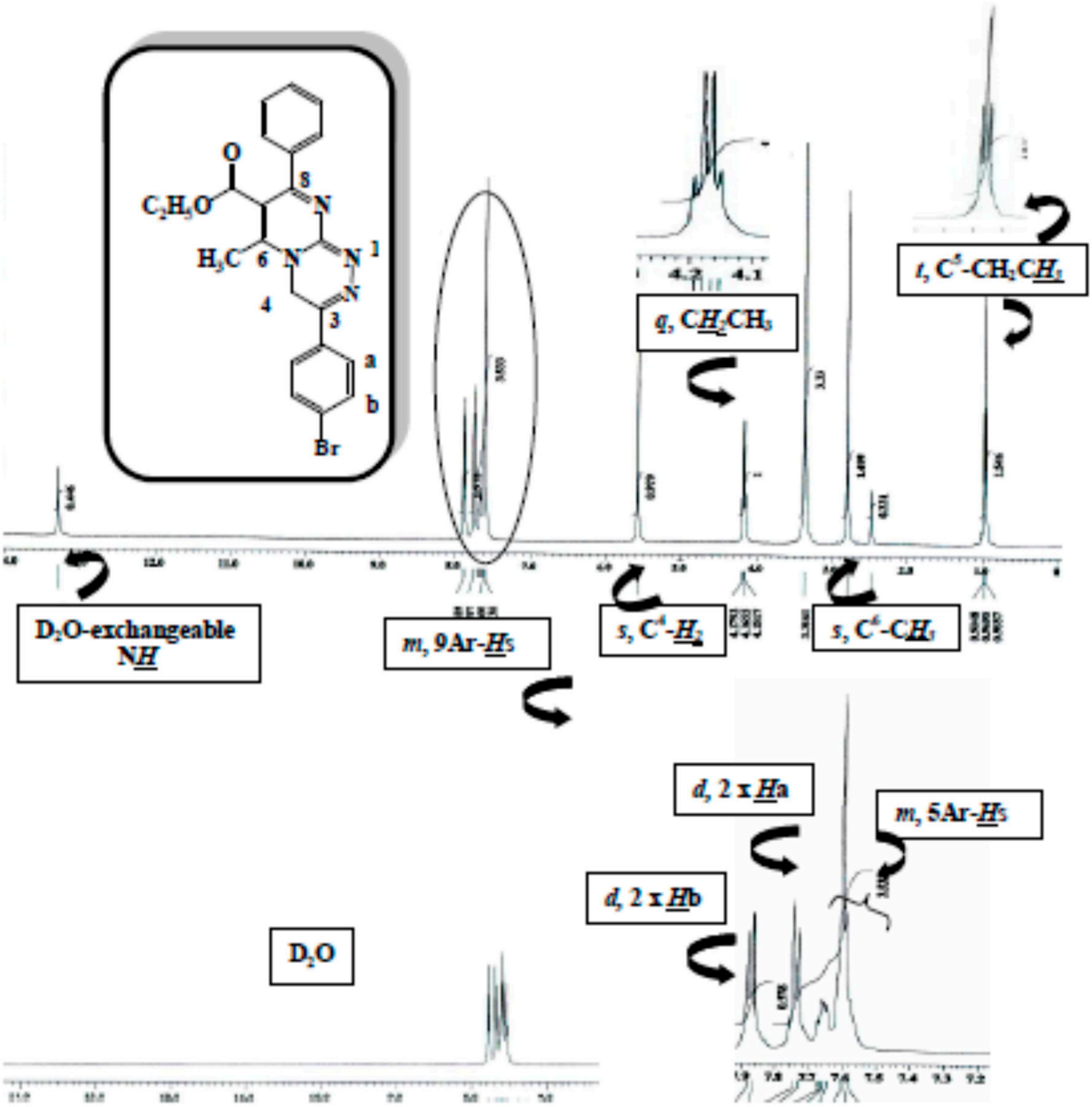

Figure S27. ^{1}H NMR spectrum of compound **14b**

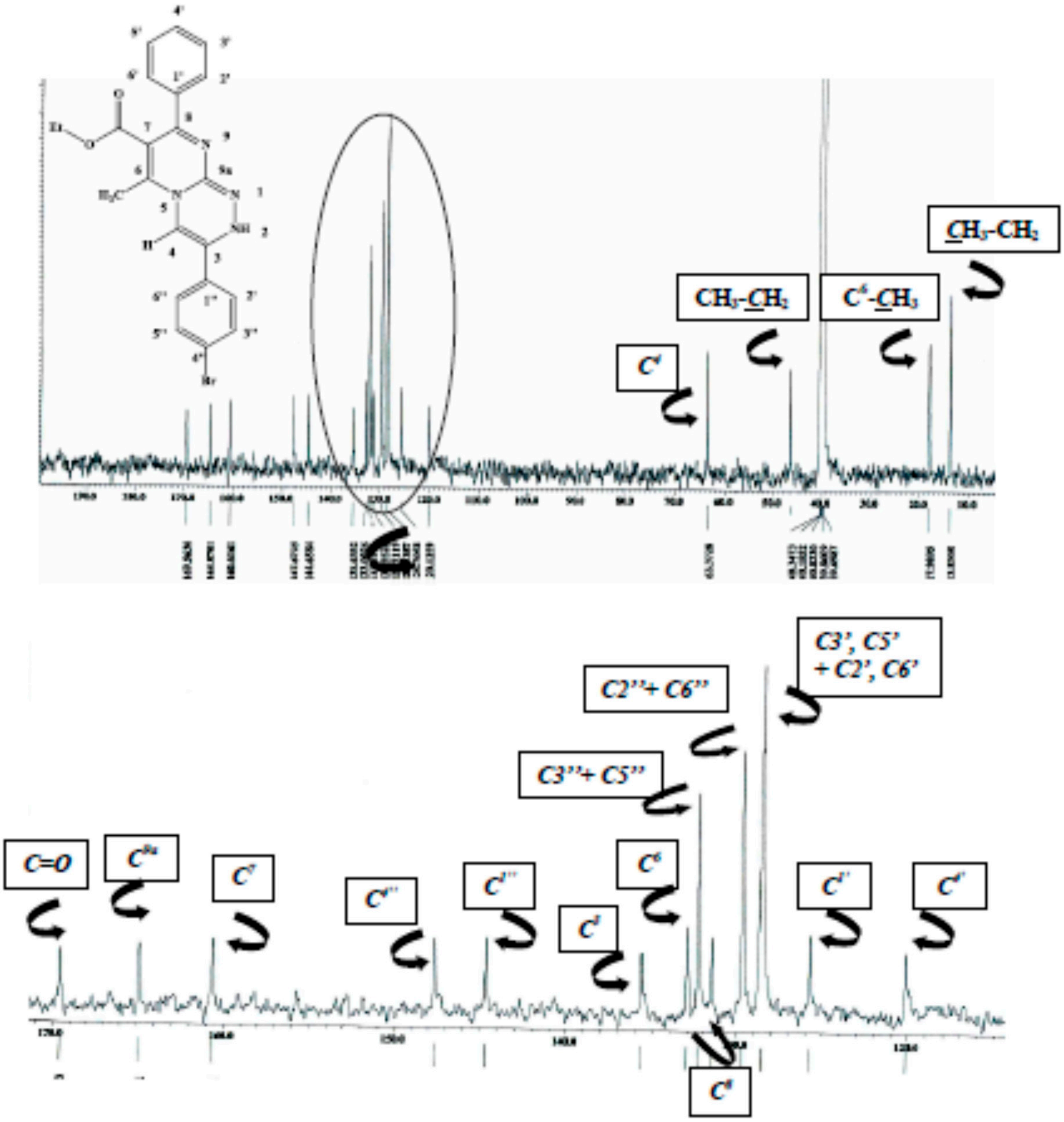

Figure S28. ^{13}C NMR spectrum of compound **14b**

Pharmacophore elucidation

- ***Selection of the Training Set Compounds:***

Selection of the training set is a key step in automatic pharmacophore generation. It governs the quality of resultant pharmacophore models and plays a critical role in the whole process. Herein, a Training set compounds consisting of nifedipine, DHPMs [1-3] (with possible tautomers) and pyrimidine-based CCBs [4] **(Figure 29; I-VII),** representing the most interesting CCBs, were selected for constructing the pharmacophore model.

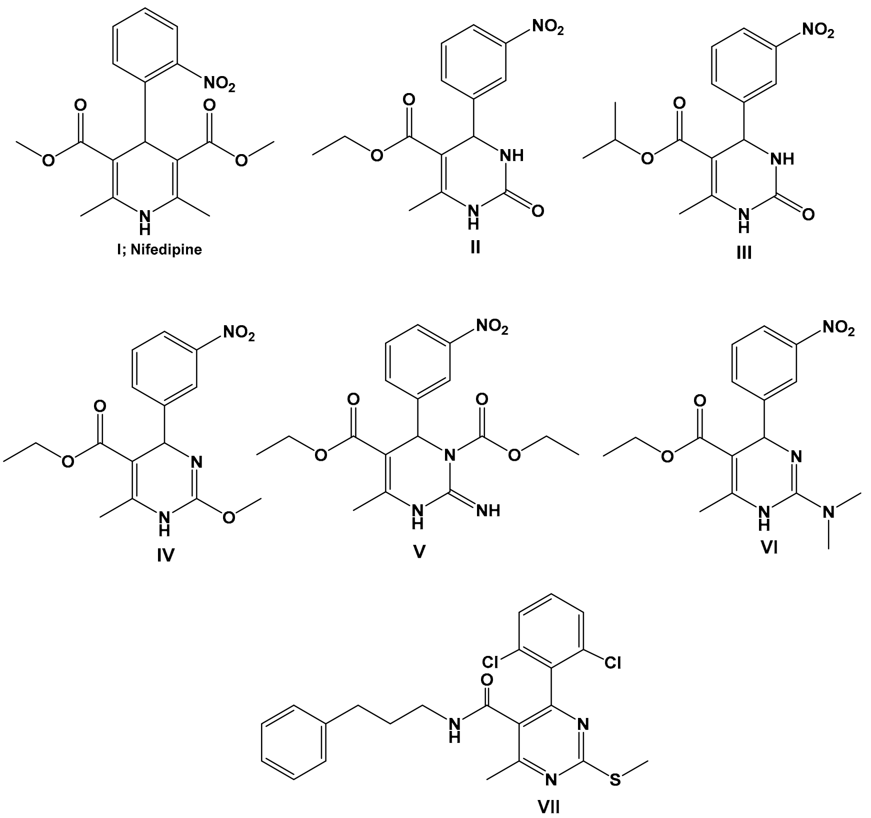

Figure S29: Training set compounds comprising representative DHP, DHPMs and pyrimidine-based CCBs

- ***Validation of the Pharmacophore Model:***

Selected pharmacophore model was validated for its predictive efficacy as calcium channel model utilizing representative derivatives of DHPs (felodipine), DHPMs [2] and pyrimidines [4] CCBs **(Figure 30).** Mapping these compounds onto the generated query **(Figure 31)** showed high mapping scoring quality expressed in terms of RMSD values. RMSD is defined as the root mean square distance between query features and their matching annotation points. Hence, the lower the RMSD values the better the compound fitness to the generated hypothesis.

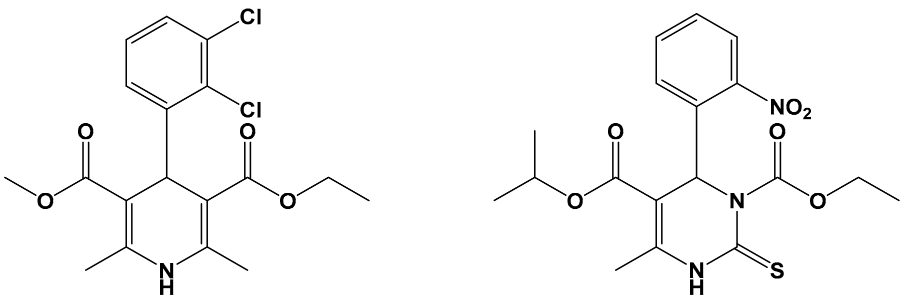

Felodipine (RMSD=0.2241 Å) **DHPM CCB (RMSD=0.5778 Å)**

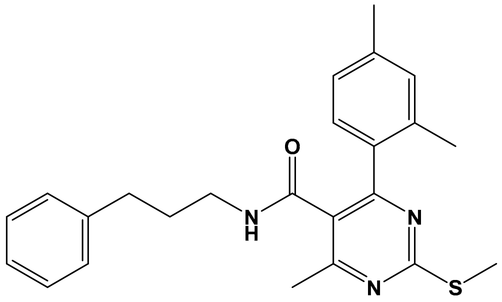

Pyrimidine-based CCB (RMSD=0.5212 Å)

Figure S30: Validation set compounds

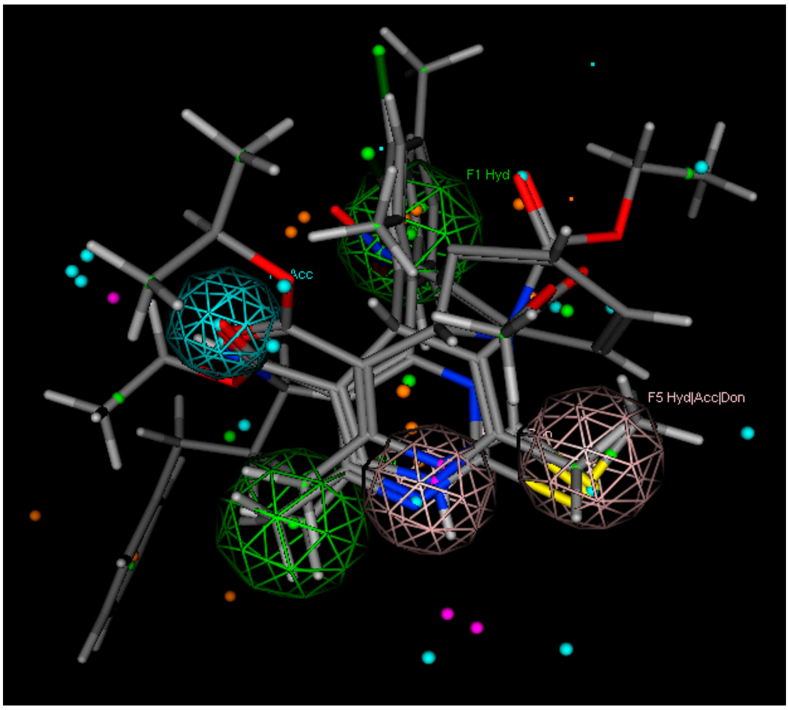

Figure S31: Mapping of validation set compounds on the pharmacophore model

References

1. Atwal, K.S.; Rovnyak, G.C.; Schwartz, J.; Moreland, S.; Hedberg, A.; Gougoutas, J.Z.; Malley, M.F. and Floyd, D.M. Dihydropyrimidine calcium channel blockers: 2-heterosubstituted 4-aryl-1, 4-dihydro-6-methyl-5-pyrimidinecarboxylic acid esters as potent mimics of dihydropyridines. *J. Med. Chem.,* **1990,** *33*, 1510-1515.
2. Atwal, K.S.; Rovnyak, G.C; Kimball, S.D.; Floyd, D.M.; Moreland, S.; Swanson, B.N.; Gougoutas, J.Z.; Schwartz, J.; Smillie, K.M. and Malley, M.F. Dihydropyrimidine calcium channel blockers. II. 3-Substituted-4-aryl-1, 4-dihydro-6-methyl-5-pyrimidinecarboxylic acid esters as potent mimics of dihydropyridines. *J. Med. Chem.*, **1990,** *33*, 2629-2635.
3. Atwal, K.S.; Swanson, B.N.; Unger, S.E.; Floyd, D.M.; Moreland, S.; Hedberg, A. and O'Reilly, B.C. Dihydropyrimidine calcium channel blockers. 3. 3-Carbamoyl-4-aryl-1, 2, 3, 4-tetrahydro-6-methyl-5-pyrimidinecarboxylic acid esters as orally effective antihypertensive agents. *J. Med. Chem.*, **1991,** *34*, 806-811.
4. Ohno, S.; Otani, K.; Niwa, S.; Iwayama, S.; Takahara, A.; Koganei, H.; Ono, Y.; Fujita, S.; Takeda, T.; Hagihara, M. and Okajima, A.; Int. Patent Appl. WO 2002022588 (2002).
